# A Radical Solution: The Phylogeny of the Nudibranch Family Fionidae

**DOI:** 10.1371/journal.pone.0167800

**Published:** 2016-12-15

**Authors:** Kristen Cella, Leila Carmona, Irina Ekimova, Anton Chichvarkhin, Dimitry Schepetov, Terrence M. Gosliner

**Affiliations:** 1 Department of Invertebrate Zoology, California Academy of Sciences, San Francisco, California, United States of America; 2 Department of Marine Sciences, University of Gothenburg, Gothenburg, Sweden; 3 Far Eastern Federal University, Vladivostok, Russia; 4 Biological Faculty, Moscow State University, Moscow, Russia; 5 A.V. Zhirmunsky Instutute of Marine Biology, Russian Academy of Sciences, Vladivostok, Russia; 6 National Research University Higher School of Economics, Moscow, Russia; University of California, UNITED STATES

## Abstract

Tergipedidae represents a diverse and successful group of aeolid nudibranchs, with approximately 200 species distributed throughout most marine ecosystems and spanning all biogeographical regions of the oceans. However, the systematics of this family remains poorly understood since no modern phylogenetic study has been undertaken to support any of the proposed classifications. The present study is the first molecular phylogeny of Tergipedidae based on partial sequences of two mitochondrial (COI and 16S) genes and one nuclear gene (H3). Maximum likelihood, maximum parsimony and Bayesian analysis were conducted in order to elucidate the systematics of this family. Our results do not recover the traditional Tergipedidae as monophyletic, since it belongs to a larger clade that includes the families Eubranchidae, Fionidae and Calmidae. This newly recovered clade is here referred to as Fionidae, the oldest name for this taxon. In addition, the present molecular phylogeny does not recover the traditional systematic relationships at a generic level, and therefore, systematic changes are required. We recognize the following clades within Fionidae: *Calma*, *Cuthona*, *Cuthonella*, *Eubranchus*, *Fiona*, *Murmania*, *Tenellia*, *Tergipes*, *Tergiposacca* gen. nov., *Rubramoena* gen. nov. and *Abronica* gen. nov. The type species of *Tergiposacca*, *T*. *longicerata* nov. sp. is described. The other two new genera have a previously described species as their type species. Most of these taxa, with the exceptions of *Eubranchus*, *Tergipes* and *Fiona* are composed of radically different constituent species from their traditional membership, but appear to be supported by morphological synapomorphies as well as molecular data. *Aenigmastyletus*, *Catriona*, *Phestilla*, *Tenellia* and *Trinchesia* are nested within other clades and, thus are here considered as synonyms of the larger clades. The phylogenetic position and validity of *Myja*, *Guyvalvoria*, *Leostyletus* and *Subcuthona* still need to be tested in future studies when material becomes available.

## Introduction

Found in nearly every ocean worldwide [1], members of the aeolid family Tergipedidae Bergh, 1889 represent a truly successful group of nudibranchs. In the Indo-Pacific alone, there are over seventy tergipedid species most of which are undescribed [2]. Nearly all tergipedid species feed exclusively upon hydroids that often settle on submerged stationary and floating objects (e.g., [1, 3]), but some other members of this family represent the only aeolid lineage that feeds on scleractinian corals [4]. Consequently, tergipedids have been found to inhabit most marine ecosystems, from the Indo-Pacific tropics, throughout temperate waters, to the Artic and Antarctica [1, 4–13]. This wide geographic distribution, however, has hindered researchers attempts at identifying and classifying tergipedid species and has led to numerous synonymous descriptions and undescribed species [5, 14–18]. After much reorganization [3, 14–15], Tergipedidae currently consists of approximately 110 species [19] unevenly included in eight to eleven genera, depending on the author [3, 14, 19] (Table 1). Some of these genera are monotypic: *Tenellia* A. Costa, 1866, which has a velum instead of cephalic tentacles [20]; *Subcuthona* Baba, 1949, with simple digestive ducts, single ceras per row and the precardiac part of the duct system with more than one diverticulum [3]; *Murmania* Martynov, 2006, characterized by lateral tooth denticles forming clusters [11]; and *Myja* Bergh, 1896, which is very similar to *Tergipes*, but possesses cerata arranged in small groups [21]. However, the status of the latter genus within Tergipedidae is doubtful [19]. Most tergipedid genera include more than one species. *Tergipes* Cuvier, 1805, the type genus of Tergipedidae, is defined by having simple digestive ducts, single ceras per row and the precardiac part of the duct system with only one diverticulum [3]. The genus *Phestilla* Bergh, 1874 represents several coral-eating tergipedid species with cephalic tentacles greatly reduced and lacking cnidosacs at the tips of the cerata [3, 20]. *Trinchesia* Ihering, 1879, originally distinguished from *Cuthona* Alder and Hancock, 1855 by the possession of a penial stylet, is regarded by most authors to be a synonym of *Cuthona* [3, 14]. The four species that form *Cuthonella* Bergh, 1884, are characterized by having the digestive ducts branched, with a single row of diverticula, and the penial gland connected with the vas deferens [3, 22]. The Antarctic genus *Guyvalvoria* Vayssière, 1906, is constituted by only four species that are unique in Tergipedidae, since their anus is not surrounded by cerata [11]. A few species have been placed in *Catriona* Winkworth, 1941, whose distinct radula and jaw morphologies arguably distinguish them from other tergipedids [14, 20]. Finally, the remaining majority of tergipedid species fall into *Cuthona*, the “catch-all” genus of Tergipedidae.

**Table 1:** Accepted genera of Tergipedidae based on bibliography.

	*Tergipes*, Cuvier 1805	*Cuthona* Alder and Hancock, 1855	*Tenellia* A. Costa, 1866	*Phestilla* Bergh, 1874	*Cuthonella* Bergh, 1884	*Trincehsia* Inhering, 1879	*Ennoia* Bergh, 1896	*Myja* Bergh, 1896	*Zatteria* Eliot, 1902	*Guyvalvoria* Vayssière, 1906	*Precuthona* Odhner, 1929	*Cratenopsis* Lemche, 1935	*Indocratena* Odhner, 1940	*Catriona* Winckworth, 1941	*Subcuthona* Baba, 1949	*Murmania* Martynov, 2006
Miller, 1977	X	X	X	X	X		?	?	?	X	X	?	?		X	
Williams and Gosliner, 1979	X	X	X	X	X					X	X	?	?	X	X	
Bouchet, 2014	X	X	X	X	X	X		?		X						X

Abbreviations, X: genus accepted by the author; ?: doubtful genus.

In the last few decades, *Cuthona*, *Catriona* and *Trinchesia* have been used both separately and interchangeably by various authors (Table 2). Most authors debated whether these three genera possess enough inter-specific anatomical variation for making generic distinctions [3, 14, 15, 20, 23–25].

**Table 2:** Previous classifications of *Catriona*, *Cuthona* and *Trinchesia*.

PUBLICATIONS	GENERA
Burn, 1973	***Catriona*** *Cuthona =* ***Trinchesia***
Miller, 1977	*Catriona =* ***Cuthona*** *= Trinchesia*
Williams and Gosliner, 1979	***Catriona Cuthona*** *= Trinchesia*
Brown, 1980	*Catriona =* ***Cuthona*** *= Trinchesia*
Miller, 2004	***Cuthona Trinchesia*** *= Catriona*

Names in bold are the proposed accepted genera and underlined names are proposed synonyms.

Miler [3] considered *Trinchesia* and *Catriona* a junior synonym of *Cuthona*, whereas Burn [20] employed *Catriona* as a distinct genus based on the presence of pre-radular teeth and bristle-like denticles along the masticatory edge of the jaw. Williams and Gosliner [14] agreed with this classification except that they recognized *Trinchesia* as a junior synonym of *Cuthona*, based on priority of the name *Cuthona*. Miller [3] and Brown [15] argued that *Catriona’s* dental morphology does not show enough distinction to warrant its separation as a distinct genus and thereby considered *Catriona* and *Trinchesia* to be junior synonyms of *Cuthona*. Miller [23] later suggested an entirely new classification by re-introducing the genus *Trinchesia* and transferring into it all tergipedid species except *Cuthona nana* (Alder and Hancock, 1842), *Cuthona*’s type species. He justified this re-organization based on the ceratal arrangement and secondary branching of the hepatic ducts that he used to define *Trinchesia*. Despite thorough morphological analyses that resulted from these controversies, no study has provided strong evidence for any of the proposed classifications and thus the systematics of Tergipedidae remains poorly understood. Furthermore, all this has been done in the absence of any proposed phylogeny.

The primary aim of this study is to provide the first molecular phylogenetic analysis of Tergipedidae, based on two mitochondrial and one nuclear gene molecular markers [partial sequences of mitochondrial Cytochrome с oxidase subunit I (COI) and 16S rRNA (16S) and nuclear Histone H3 (H3)]. The specific goal of this study is to test the monophyly of the tergipedid genera *Catriona*, *Cuthona*, *Cuthonella*, *Murmania*, *Phestilla*, *Tenellia*, *Tergipes* and *Trinchesia*, reviewing their phylogenetic relationships and to construct a new classification of the Tergipedidae, that reflects monophyly based on revised phylogenetic hypotheses.

## Materials and Methods

### Taxon sampling

Specimens were collected on recent field trips carried out by the authors as well as from specimens deposited at the California Academy of Sciences, CASIZ (San Francisco, USA), Göteborgs Naturhistoriska Museum, GNM (Gothenburg, Sweden), and at the subsidiary of Zoological Museum of Lomonosov Moscow State University at the White Sea Biological Station, WS (Primorsky, Karelia, Russia). Two hundred and thirty-six nudibranch specimens were analyzed in this study representing 9 families, 21 genera, 73 described species and 19 undescribed species. This included specimens of 55 species traditionally included in the Tergipedidae and an additional 13 taxa that have been variously ascribed to be closely related to Tergipedidae, including members of the Eubranchidae, Calmidae and Fionidae. All the species and sequences used in this study are listed in S1 Table and arranged based on the classification found in Bouchet and Rocroi [26] and Gosliner et al. [4]. Numbers following “sp.” in the names of undescribed species refer to the identification system used by Gosliner et al. [4]. Undescribed species labeled as “sp.” followed by a capital letter refer to undescribed species not included in Gosliner et al. [4]. Broad taxon sampling was necessary to ensure that the type species for each tergipedid genus was represented whenever it was available. Due to the lack of previous phylogenetic research on Tergipedidae, broad taxon sampling was also necessary to determine a suitable outgroup. Eubranchidae was the first outgroup tested based on its triseriate radula, which is thought to be a more primitive than the uniseriate radula [5]. However, preliminary analyses suggested that it was too closely related to Tergipedidae and may, in fact be part of the ingroup (not shown). Thus, *Tritonia pickensi* Marcus & Marcus, 1967 was chosen as a distant out-group, whereas the remaining closely related species of Aeolidiidae, Babakinidae, Facelinidae, Flabellinidae, Glaucidae and Tritoniidae were chosen based on the work of Pola & Gosliner [27].

Excluding *Cuthonella*, the identification of the different clades was conducted based on the type species of each genus. In addition, specimens of the type species were chosen strategically, attempting to sequence specimens from as close to the type locality as possible. For those inconclusive clades, current names were retained. In order to minimize disruption to nomenclature, synonyms were resurrected whenever possible.

### DNA extraction, amplification and sequencing

Information about the protocols followed by each author is provided as supporting information (S1 Appendix). All new sequences have been deposited in GenBank (S1 Table).

### Sequence alignment and phylogenetic analyses

Forward and reverse sequences for each gene fragment were edited, assembled and the primers were removed using Geneious 4.7.6 (Biomatters, Auckland, New Zealand). All the sequences were checked for contamination with BLAST [28]. Multiple sequence alignments were created using MAFFT [29]. The alignments were checked by eye using AliView [30]. To help verify the authenticity of the coding sequences, the protein-coding sequences for H3 and COI were translated into amino acids with AliView. Individual gene alignments were concatenated into a combined-gene dataset using Geneious 4.7.6. Saturation was visually inspected in MEGA 5.0 [31] by plotting for all specimens including outgroup the total number of pairwise differences (transitions and transversions) against uncorrected *p*-distances. For the COI and H3 fragments, saturation was further examined separately for the first, second and third codon positions.

The most variable regions from the 16S rRNA alignment were removed using the default settings in Gblocks [32]. Excluding “indel-rich” regions, the tree was in general poorly resolved with lower node support. Therefore, final analyses were performed including all bases.

Individual markers analyses and a concatenated analysis were performed. MrModeltest [33] was used to estimate the best substitution model for each partition based on Akaike information criterion. The selected model was GTR+I+G for COI and 16S, while for H3 the best model was SYM+I+G. Maximum likelihood analyses were carried out in RAxML v. 7.2.6 [34] using the GTR-GAMMAI model. Protein-coding gene datasets (COI and H3) were partitioned by codon and the combined-gene dataset was partitioned by gene and by codon. Support values were obtained by running a 5000 replicate ML “Fast Bootstrapping” [34] analysis. For Bayesian inference analyses (BI) the combined-gene dataset was partitioned by each fragment. Bayesian inference was conducted in MrBayes 3.1.2 [35]. Analyses were run for twenty five million generations with Markov chains sampled every 1000 generations. Convergence was diagnosed graphically by plotting for each run the likelihood against the number of generations using the software Tracer version 1.4.1 [36]. For each analysis the first 6250 (25%) trees were discarded (‘burn-in’ period) and node support was assessed with posterior probabilities (PP). Only nodes supported by BS ≥ 70 [37] and PP ≥ 0.95 [38] are discussed.

### Species delimitation analyses

In order to compare the genetic distances amongst specimens of Tergipedidae, we calculated the pairwise uncorrected *p*-distances for COI using PAUP* 4.0 b 10.0 [39]. All codon positions were considered for the analysis. We also applied the Automatic Barcode Gap Discovery (ABGD) method to detect species-level clusters [40]. The Automatic Barcode Gap Discovery (ABGD) method was run for COI. ABGD settings were the following: Pmin = 0.001, Pmax = 0.1, Steps = 10, X = 1.0, Nb bins = 20, and Jukes Cantor (JC69), Kimura (K80) and Simple Distance.

Poisson Tree Processes (PTP) [41] was also used for species delimitation. This approach is based on phylogenetic species concept assumption, thus implements Bayesian MCMC methods to find the groups descent from a single ancestor (i.e. phylogenetic species) using previously inferred phylogenetic tree. The test was run using the bPTP Server http://species.h-its.org/ptp/ with 500000 generations and with other settings set as default. Convergence quality was checked using ML convergence plot generated by bPTP Server. The Bayesian phylogenetic tree inferred using concatenated dataset (inferred as described above) was used as the input tree.

### Morphological studies

Morphological techniques were used to investigate unusual nodes found in the phylogenetic trees. Depending on the species in question, either the buccal mass or the reproductive system, or in some cases both, was removed and examined. Buccal masses, containing the jaws, radula and connective tissue, were carefully extracted from specimens with the aid of a dissecting microscope and forceps. The mass was placed in 10% sodium hydroxide (NaOH), allowed to soak for 4–24 hours and then rinsed in ddH20 or deionized H20. Once all connective tissue was removed, the radula and jaw were dried and mounted for examination by scanning electron microscope (SEM). Reproductive systems were carefully removed, examined and sketched under a dissecting microscope with a camera lucida. Some penial morphology was also examined by SEM.

### Ethic statements

The majority of the specimens used in this study are deposited in the collections the California Academy of Sciences Invertebrate Zoology (CASIZ) collection. We had the permission to take tissue samples from all the specimens for DNA analyses, regardless where the specimen was deposited. As stated in the CASIZ collections policy: ‘No specimens will be accessioned without adequate labeling, collection notes, field notes, or other locality information, nor without appropriate legal documentation (collecting permits, export permits from country of origin, etc.) when applicable.’ All the institutions listed in point 2.1, from which material has been examined in the present study, have strict collection policies in order to insure compliance with all laws governing the collection and sampling of wildlife from the country of origin and were legally imported to the museum repository where they are listed. Donors are required to provide all the available information about their field studies, including collecting and export permits from country of origin, as well as the number of species and specimens exported. Therefore, the legality of all this material is assumed. On the other hand, all necessary permits were obtained for the field studies carried out in the Philippines by the authors. The collecting permits were issued by the Bureau of Fisheries and Aquatic Resources (BFAR), the agency that has jurisdiction over all marine biological collections in coastal waters of the Philippines. We received Prior Informed Consent from all relevant local municipalities, a Gratuitous Permit from BFAR to permit collection of specimens and a Commodity Clearance from BFAR to permit legal exportation from the Philippines as well as clearance from the United States Fish and Wildlife Service confirming they were legally exported from the host country and legally imported into the U.S. For the remaining localities, the specimens were collected and sent by several gracious collaborators. These locations were not privately-owned or legally protected. We can confirm that we received assurances from collectors that these specimens all had the necessary permits and were legally collected. They met the California Academy of Sciences' requirements for legal accessioning into the institution's collections. Finally, none of the studied species are protected, listed as endangered, or are CITES listed.

### Nomenclatural acts

The electronic edition of this article conforms to the requirements of the amended International Code of Zoological Nomenclature, and hence the new names contained herein are available under that Code from the electronic edition of this article. This published work and the nomenclatural acts it contains have been registered in ZooBank, the online registration system for the ICZN. The ZooBank LSIDs (Life Science Identifiers) can be resolved and the associated information viewed through any standard web browser by appending the LSID to the prefix "http://zoobank.org/". The LSID for this publication is: urn:lsid:zoobank.org:pub:4299DF54-AED2-450E-A7A1-CCC86471B267.

## Results

### Sequence analyses

Sequence alignment and gene concatenation yielded a combined dataset of 1464 (including variable sites) base pairs in length. We obtained over 600 new sequences; 205 for H3, 206 for COI and 210 for 16S. Substitution saturation plots revealed no saturation in any gene (not shown). The combined tree provided better resolution than H3, COI, or 16S separately (see the complete trees in S1 Fig). The COI gene better resolved the relationships at the species and generic levels, followed by 16S, while H3 did little or no resolution. Because the analyses represent unlinked genes, they can provide different gene evolutionary histories [42–44]. As in the present paper, many studies focused on Heterobranchia have showed that combined analysis can provide better-resolved trees (e.g., [45–48].

The minimum pairwise uncorrected *p*-distances for COI among key taxa and among genera, using the reviewed names, are presented in Tables 3 and 4 respectively.

**Table 3:** Minimum COI gene pairwise uncorrected *p*-distances amongst key species with names after analyses.

Taxa	COI genetic distances	
*Cuthonella marisalbi*	*Cuthonella concinna*	0%
*Cuthona nana*	*Cuthona hermitophila*	0.1%
*Tenellia lugubris*	*Tenellia sibogae*	1.8%
*Cuthona divae*	*Cuthona hermitophila*	2.2%
*Cuthona divae*	*Cuthona nana*	2.4%
*Eubranchus rupium*	*“Eubranchus rupium”*	4.9%
*Tenellia speciosa*	*“Tenellia speciosa”*	5.2%
*Cuthonella concinna*	*Cuthonella cocoachroma*	11.4%
*Tenellia* sp. E	*Tenellia* sp. F	12.3%
*Calma glaucoides*	*Calma gobioophaga*	12.5%
*Tenellia caerulea* Spain	*Tenellia caerulea* Ireland	12.5%
*Tenellia pustulata*	*“Tenellia pustulata”*	13%

**Table 4:** Minimum COI gene pairwise uncorrected *p*-distances amongst Fionidae genera.

	*Calma*	*Cuthona*	*Cuthonella*	*Eubranchus*	*Fiona*	*Murmania*	*Tenellia*	*Tergipes*	*Abronica* gen. nov.	*Tergiposacca* gen. nov.	*Rubramoena* gen. nov.
*Calma*	0	-	-	-	-	-	-	-	-	-	-
*Cuthona*	18.4	0	-	-	-	-	-	-	-	-	-
*Cuthonella*	14.4	17.3	0	-	-	-	-	-	-	-	-
*Eubranchus*	16	16.8	14.7	0	-	-	-	-	-	-	-
*Fiona*	20	19.3	18.23	15.8	0	-	-	-	-	-	-
*Murmania*	17.3	16.7	15.5	15.6	17.4	0	-	-	-	-	-
*Tenellia*	14.7	16.9	13.7	13	16.4	15.7	0	-	-	-	-
*Tergipes*	13.4	18.4	16.8	16.9	16.7	18	17.9	0	-	-	-
*Abronica* gen. nov.	17.6	18.8	16.3	16	17.3	17.4	16.6	18.4	0	-	-
*Tergiposacca* gen. nov.	16.4	20.8	17.9	16.4	17.4	19.8	18.9	18.6	17.7	0	-
*Rubramoena* gen. nov.	16.9	17.4	14.1	13.6	18	17	13	17.2	19.4	17.8	0

### Phylogeny at a family level

When *Tergipes antarcticus* Pelseneer, 1903 was included in the analyses, the topologies of the ML trees were not congruent with the results yielded by the Bayesian analyses. Maximum likelihood analyses recovered this species nested among *Eubranchus* species, but with no support (ML = 17, S2A Fig), while in the Bayesian inference *T*. *antarcticus* was sister to the remaining specimens of Tergipedidae (PP = 1, S2B Fig). This disagreement could be because of only a single gene sequence (COI) from GenBank was available (see S1 Table). Since the identity of the specimen could not be reviewed or confirmed nor additional genes obtained, final analyses excluded this taxon.

**Fig 1:**
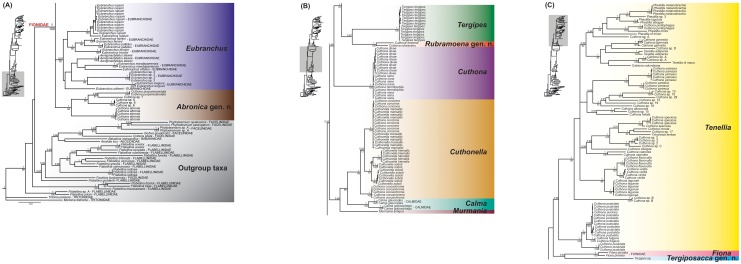
Phylogenetic hypothesis based on the combined dataset (H3+COI+16S) inferred by Bayesian analysis (BI). Support values shown represent posterior probabilities from Bayesian inference and bootstrap values from Maximum likelihood (PP/ML). **Referred to as *Cuthona* sp. 13 in Pittman and Fiene [49].

**Fig 2:**
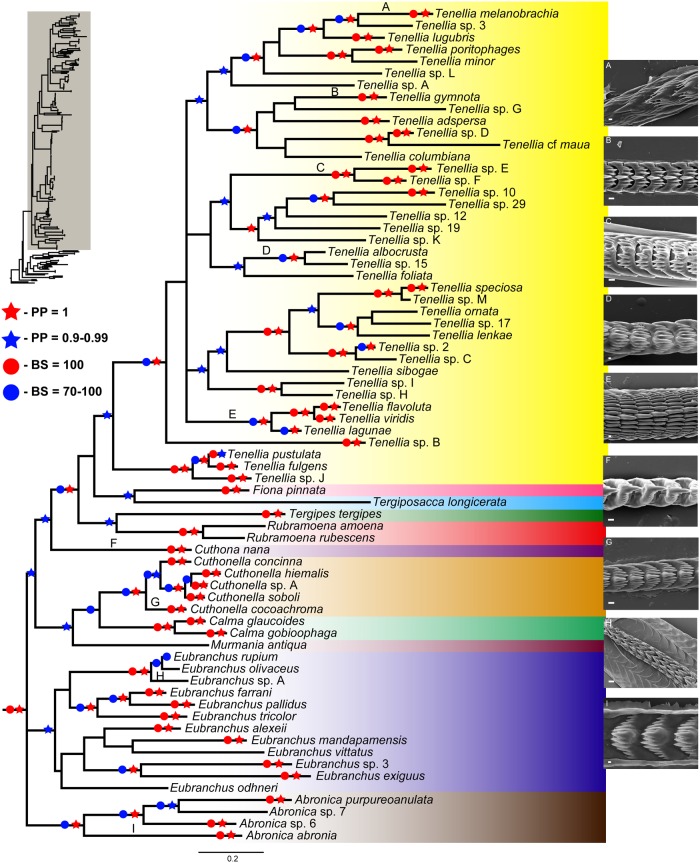
Molecular phylogeny based on the combined dataset (H3+COI+16S) inferred by Bayesian analysis (BI). Only one specimen per species of Fionidae is shown. Support values shown represent posterior probabilities from Bayesian inference and bootstrap values from Maximum likelihood (PP/ML). Diversity of fionid radula morphology is also mapped. (A) *Tenellia melanobrachia* (CAS 177299), scale bar = 10 μm. (B) *Tenellia gymnota* (CAS 68909b), scale bar = 50 μm. (C) *Tenellia yamasui* (CAS 176738), scale bar = 50 μm. (D) *Tenellia albocrusta* (CAS 71754), scale bar = 3 μm. (E) *Tenellia lagunae* (CAS 71383), scale bar = 3 μm. (F) *Cuthona nana* (CAS 87192), scale bar = 50 μm. (G) *Cuthonella cococachroma* (CAS 71755a), scale bar = 50 μm. (H) *Eubranchus* sp. (CAS 114833), scale bar = 50 μm. (I) *Abronica abronia* (CAS 71753), scale bar = 3 μm.

Without *T*. *antarcticus*, the topologies of the ML trees were completely concordant, with the results obtained by Bayesian inference, although bootstrap values were lower than posterior probabilities in larger clades. In addition, both ML and Bayesian PTP analyses supported delimitation of all studied species present on Figs 1 and 2 (S3 Fig). Figs 1 and 2 show the resulting phylogenetic hypothesis based on the combined dataset represented by BI. Fig 1 depicts species names with the preliminary identifications, whereas Fig 2 excludes the whole outgroup and shows only one representative of each species with the final revised names. Our analyses rejected the monophyly of Tergipedidae, as it has been traditionally defined. Instead, Eubranchidae, Fionidae and Calmidae are consistently nested within the tergipedids. Together, these four families constituted a clade with a high support in all analyses (PP = 1, ML = 100). The 16S trees also supported this relationship (PP = 1, ML = 97) (S1C Fig). The COI also recovered this relationship, but it was not supported (PP = 0.80, ML = 65) (S1B Fig). However, the H3 did not show any resolution at a family level (S1A Fig).

Eubranchidae appears as monophyletic (PP = 0.97, ML = 62), with representatives of two genera, *Eubranchus* Forbes, 1838 and *Aenigmastyletus* Martynov, 1998. Relationships among the different species included in this study were not resolved, as this was not the focus of this study. However, the type species, *Eubranchus tricolor* Forbes, 1838 was closely related with *E*. *farrani* (Alder and Hancock, 1844) and *E*. *pallidus* (Alder and Hancock, 1842) (PP = 0.99, ML = 51). In addition, the specimens of *E*. *rupium* (Møller, 1842) included in this study formed three different groups, with a minimum uncorrected *p*-distance of 4.9% for COI among groups. ABGD analyses revealed the likely existence of cryptic diversity within this clade (PP = 1, ML = 100). Independently of the chosen model (Jukes-Cantor, Kimura or Simple Distance), these analyses recovered from two to five groups, with a P values ranging from 0.001 to 0.036 (S4A Fig).

The two species of Calmidae, *Calma glaucoides* (Alder and Hancock, 1854) and *C*. *gobioophaga* Calado and Urgorri, 2002 were sister to each other and formed a high supported clade (PP = 1, ML = 100) (minimum uncorrected *p*-distance = 12% for COI between both species).

The monospecific family Fionidae appeared to be closely related to an undescribed species of *Tergipes*, but this relationship was only supported by Bayesian inference (PP = 0.93, ML = 45).

#### Phylogeny at the “generic level”

Relationships among the different genera traditionally attributed to Tergipedidae were also not clearly recovered as monophyletic. *Cuthona* is polyphyletic although most *Cuthona* species included in this study clustered together in a large clade (PP = 0.98, ML = 54). Within this major clade, two undescribed species, erroneously identified as *Cuthona yamasui* Hamatani, 1993 [4] appeared here as not one, but two distinct sister species ([2]: 345, as *Cuthona* sp. 13 and *Cuthona* sp. 14) that are sympatric in the Philippines and Malaysia (minimum uncorrected *p*-distance for COI between clades 12.3%). ABGD analyses recovered from two to three groups (putative species), independently of the chosen model (Jukes-Cantor, Kimura or Simple distance), with a P values ranging from 0.001 to 0.1 (S4B Fig). Puillandre et al. [40] concluded that a higher number of groups are expected with lower P values, in which case, the method suggests less realistic species hypotheses scenarios. Therefore only those groups with the highest P value will be represented (S4 Fig). The uncorrected *p*-distance for COI between the two specimens of *Cuthona caerulea* (Montagu, 1804) was 12.5%, suggesting the existence of a species complex. In this case, ABGD could not be applied because of the existence of only two specimens. The four specimens of *Cuthona speciosa* formed two subclades (PP = 1, ML = 100) (minimum uncorrected *p*-distance of between the specimen CASIZ 176185 and the remaining specimens = 5.2%) and likely include two different species, although ABGD analyses did not recover these two groups. In addition, the type species of *Cuthona*, *Cuthona nana*, formed a separated clade together with all the specimens identified as *Cuthona divae* (Er. Marcus, 1961) and *Cuthona hermitophila* Martynov, 2015 (PP = 1, ML = 100), being the maximum uncorrected *p*-distance of among species was 3.3%. ABGD analyses did not find significant difference between these specimens. The specimens initially identified as *C*. *pustulata* were divided into two separate clades, both with high support (minimum uncorrected *p*-distance between them = 13%). The smallest one was formed by three specimens from the Barents Sea (PP = 1, ML = 100), whereas specimens from Maine, the Barents Sea, the White Sea plus one specimen identified as *Cuthona punicea* Millen, 1986 from Canada (PP = 1, ML = 100) constituted the largest clade. ABGD analyses found from two to four groups for Jukes-Cantor model and Simple Distance, and from two to three groups for Kimura (P values from 0.001 to 0.1 for the three models) (S4C Fig). *Cuthona fulgens* (MacFarland, 1966) was the sister species of these two clades.

*Catriona* alone was not monophyletic, but constituted a strongly supported (PP = 1, ML = 95) clade with the inclusion of *Cuthona* sp. D (named as *Cuthona* sp. 13 in Pittman and Fiene [49]) and *Tenellia adspersa* (Nordmann, 1845). In addition, the two western Atlantic specimens of *Catriona gymnota* (Couthouy, 1838) formed a separately clade (PP = 0.99, ML = 100) from the European specimen (PP = 1, ML = 100). The branch length may indicate that both populations are not conspecific, but neither *p*-distance nor ABDG analyses were conducted due to the lack of COI sequence from the Swedish specimen.

*Cuthona poritophages* Rudman, 1979, which also feeds on scleractinean corals, and *Cuthona* sp. A clustered among *Phestilla* species (PP = 0.94, ML = 59). The *p*-distance between *Phestilla lugubris* (Bergh, 1870) and *P*. *sibogae* Bergh, 1905 was only 1.8% and these two species have been regarded as synonyms [16, 50].

Although the type species of *Cuthonella* was not available for this study, *C*. *marisalbi* (Roginskaya, 1963), *C*. *soboli* Martynov, 1992 and *C*. *hiemalis* (Roginskaya, 1987), clustered in a single clade together with *Cuthona cocoachroma* Williams and Gosliner, 1979 and *C*. *concinna* (Alder and Hancock, 1843) (PP = 1, ML = 98). The latter two species had between them an uncorrected *p*-distance of 11.3% (COI). This separation was supported by the ABGD analyses, which recovered two groups with a P values ranging from 0.001 to 0.1 (S4D Fig) (Jukes-Cantor, Kimura and Simple Distance models) and PTP analysis that found 94% and 96% delimitation support for these species, respectively (S3 Fig). The *Cuthonella marisalibi* specimens were nested with the same clade as the *Cuthona concinna* (PP = 0.99, ML = 100) (maximum uncorrected *p*-distance = 2.6% for COI) specimens. ABGD analyses recovered from one to two groups (independently of the model), with a P values ranging from 0.001 to 0.0046 (S4D Fig), whereas PTP analysis revealed 94% support for single species hypothesis. In addition, those specimens identified as *Cuthonella soboli* were separated into two clades with maximum support (PP = 1, ML = 100 both of them). Two specimens from Kamchatka constituted the smallest clade, and the minimum uncorrected *p*-distance for COI between the latter and the other clade was of 7.7%. ABGD and PTP analyses also found cryptic diversity among these specimens since they recovered two groups with 100% support by PTP (S3 Fig) and Simple Distance, and from three to five groups for Jukes-Cantor and Kimura, with a P values ranging from 0.001 to 0.06 for the three models (S4D Fig).

*Murmania antiqua* Martynov, 2006 was sister to the latter clade plus Calmidae (PP = 0.99, ML = 57).

*Tergipes*, as well, was not recovered as monophyletic since *Tergipes* sp. was found in a clade outside of the larger clade to which *T*. *tergipes* belongs. *Cuthona amoena* (Alder and Hancock, 1845) and *C*. *rubescens* Picton and Brown, 1978 clustered together (PP = 1, ML = 100) and appeared to be closely related to *Tergipes tergipes* (Forsskål in Niebuhr, 1775) (PP = 0.97, ML = 40).

Finally, *Cuthona abronia* (MacFarland, 1966), *C*. *purpureoanulata* (Baba, 1961), *Cuthona* sp. 7 and *Cuthona* sp. 6 constituted a clade with high support (PP = 0.99, ML = 94).

## Discussion

### Tergipedidae

The primary purpose of this study is to use molecular data to establish a preliminary phylogeny of Tergipedidae, determine its monophyly and the phylogenetic status the taxa traditionally regarded as genera. However, our results encompass more than just Tergipedidae. The molecular analysis reveals that the traditional Tergipedidae is polyphyletic and belongs to a larger monophyletic clade including members of the traditional “families” Eubranchidae, Fionidae and Calmidae. The inclusion of these taxa within Tergipedidae was an unexpected result, since the validity of these taxa and their distinctness from the Tergipedidae has never been questioned. Due to fact that the Fionidae is the oldest available family group name for these taxa, we propose to unite Tergipedidae, Eubranchidae, Calmidae and Fionidae under the name of Fionidae (Fig 2), in accordance to the provisions of ICZN [51] article 23. Fionidae has several morphological synapomorphies. The anus is situated immediately anterior to the dorsal cerata of postcardiac digestive branch (acleioproctic). The head is circular in shape with rounded rather than tentacular extensions of the anterior portion of the foot. The ovotestis follicles are arranged with a series of small male acini found around the periphery of large female acini. The penis usually has always has a distinct penial gland at the posterior end of the penis.

Within Fionidae, the results presented here demonstrate the necessity of a developing a new classification to reflect the recovered clades and preserving monophyly. Previous classifications, separating *Catriona*, *Cuthona* and *Trinchesia* as distinct taxa, are all inconsistent with the resulting phylogeny [3, 14]. Our molecular data shows that the traditional membership of *Cuthona* to be paraphyletic within Fionidae and therefore suggests that under Miller’s [23] classification, in which he transfers to *Trinchesia* all the species of *Cuthona* except *Cuthona nana*, *Trinchesia* would also be paraphyletic. However, whether *Trinchesia* is distinct from *Cuthona* is not as easy to decipher. Initially, it appears that *Trinchesia* is not synonymous with *Cuthona* considering that the type species of *Trinchesia*, *Doris caerulea* Montagu, 1804, does not group with *Eolis nana* Alder and Hancock, 1842, the type species of *Cuthona*. Instead, *T*. *caerulea* groups together with most *Cuthona* species in a large clade (PP = 0.98, ML = 54). This result also confirms that a penial stylet may be either present or absent. Presence of a penial stylet was the original character used to distinguish *Trinchesia* from *Cuthona* [3, 52–54], and this distinction appears to have no relevance based on our recovered phylogeny. The presence or absence of this character is variable throughout all fionids, either evolving multiple times or has been lost multiple times, and cannot be used as a valid generic distinction for *Trinchesia*, or any other higher taxon within the Fionidae, for that matter (see below).

Prior to this study, the distinctness of the genus *Catriona* was the subject of considerable debate. Burn [20], Williams and Gosliner [14] and Gosliner and Griffiths [55] all suggested that *Catriona*’s bristled denticles and pre-radular teeth are sufficiently to designate it as a unique genus. Miller [3, 23] and Brown [15] had a different interpretation and considered the bristles an unsatisfactory diagnostic character, referring to *Catriona* as a junior synonym of *Cuthona*. Results from this study demonstrate that *Catriona* only becomes monophyletic with the inclusion of *Tenellia adspersa* and *Cuthona* sp. D (named as *Cuthona* sp. 13 in Pittman and Fiene [49]). In examining the morphology of *Cuthona* sp. D, the radula does posses a recessed median cusp (not shown), similar to other *Catriona* species. However, *Tenellia adspersa* does not possess bristles on its masticatory edge, nor does it have a recessed median cusp or pre-radular teeth. This suggests that these variations in radula and jaw morphology are not indicative of generic distinction for these taxa, and similar to the penial stylet, vary throughout all of Fionidae. Alternatively, if the taxa united by the radular and jaw features were considered as distinct genus that is sister to *Tenellia*, creation of multiple new genera would be required to achieve uniformity as to where in the tree sister taxa are considered as genera. Similar issues are evident if all the coral-eating fionids are united in a monophyletic *Phestilla* that includes *Cuthona poritophages*.

In order to recover the fionid genera that represent clades, we here propose a new classification based on our phylogenetic hypothesis and where morphology permits diagnosis of clades that are based on molecular data (Table 5).

**Table 5:** New classification of Fionidae based on monophyletic groups, including nominal species studied in the present contribution.

Systematics	Synonymized taxa	Nominal species
Family FIONIDAE Alder & Hancock, 1855	Calmidae Iredale & O’Donoghue, 1923 syn. nov.	
	Tergipedidae Thiele, 1931 syn. nov.	
	Eubranchidae Odhner, 1934 syn. nov.	
	Cuthonidae Odhner, 1934 syn. nov.	
	Trinchesiidae Nordsieck, 1972 syn. nov.	
*Tergipes* Cuvier, 1805		*Tergipes tergipes*
*Eubranchus* Forbes, 1838	*Aenigmastyletus* Martynov, 1998 syn. nov.	*Eubranchus alexeii*
		*Eubranchus exiguus*
		*Eubranchus farrani*
		*Eubranchus mandapamensis*
		*Eubranchus odhneri*
		*Eubranchus pallidus*
		*Eubranchus rupium*
		*Eubranchus olivaceus*
		*Eubranchus tricolor*
		*Eubranchus vittatus*
		*Eubranchus* sp. 3
		*Eubranchus* sp. A
*Fiona* Alder & Hancock, 1851		*Fiona pinnata*
*Calma* Alder & Hancock, 1855		*Calma glaucoides*
		*Calma gobioophaga*
*Cuthona* Alder & Hancock, 1855		*Cuthona nana*
*Tenellia* A. Costa, 1866	*Catriona* Winckworth, 1941	*Tenellia adspersa*
	*Cuthona* Alder & Hancock, 1855 (partim)	*Tenellia columbiana*
	*Phestilla* Bergh, 1874	*Tenellia flavovulta*
	*Trinchesia* Ihering, 187	*Tenellia foliata*
		*Tenellia fulgens*
		*Tenellia gymnota*
		*Tenellia lagunae*
		*Tenellia lenkae*
		*Tenellia lugubris*
		*Tenellia* cf *maua*
		*Tenellia melanobrachia*
		*Tenellia minor*
		*Tenellia ornata*
		*Tenellia poritophages*
		*Tenellia* cf *pustulata*
		*Tenellia sibogae*
		*Tenellia speciosa*
		*Tenellia viridis*
		*Tenellia* sp. 2
		*Tenellia* sp. 3
		*Tenellia* sp. 10
		*Tenellia* sp. 12
		*Tenellia* sp. 15
		*Tenellia* sp. 17
		*Tenellia* sp. 19
		*Tenellia* sp. 29
		*Tenellia* sp. A
		*Tenellia* sp. B
		*Tenellia* sp. C
		*Tenellia* sp. D
		*Tenellia* sp. E
		*Tenellia* sp. F
		*Tenellia* sp. G
		*Tenellia* sp. H
		*Tenellia* sp. I
		*Tenellia* sp. J
		*Tenellia* sp. K
		*Tenellia* sp. L
		*Tenellia* sp. M
*Cuthonella* Bergh, 1884	*Cuthona* Alder & Hancock, 1855 (partim)	*Cuthonella cocoachroma*
		*Cuthonella concinna*
		*Cuthonella hiemalis*
		*Cuthonella soboli*
		*Cuthonella* sp. A
*Myja* Bergh, 1896		
*Guyvalvoria* Vayssière, 1906		
*Subcuthona* Baba, 1949		
*Leostyletus* Martynov, 1998		
*Murmania* Martynov, 2006		*Murmania antiqua*
*Abronica* gen. nov	*Cuthona* Alder & Hancock, 1855 (partim)	*Abronica abronia*
		*Abronica purpureoanulata*
		*Abronica* sp. 6
		*Abronica* sp. 7
*Tergiposacca* gen. nov		*Tergiposacca longicerata*
*Rubramoena* gen. nov	*Cuthona* Alder & Hancock, 1855 (partim)	*Rubramoena amoena*
		*Rubramoena rubescens*

### *Calma* Alder and Hancock, 1855

**Fig 3:**
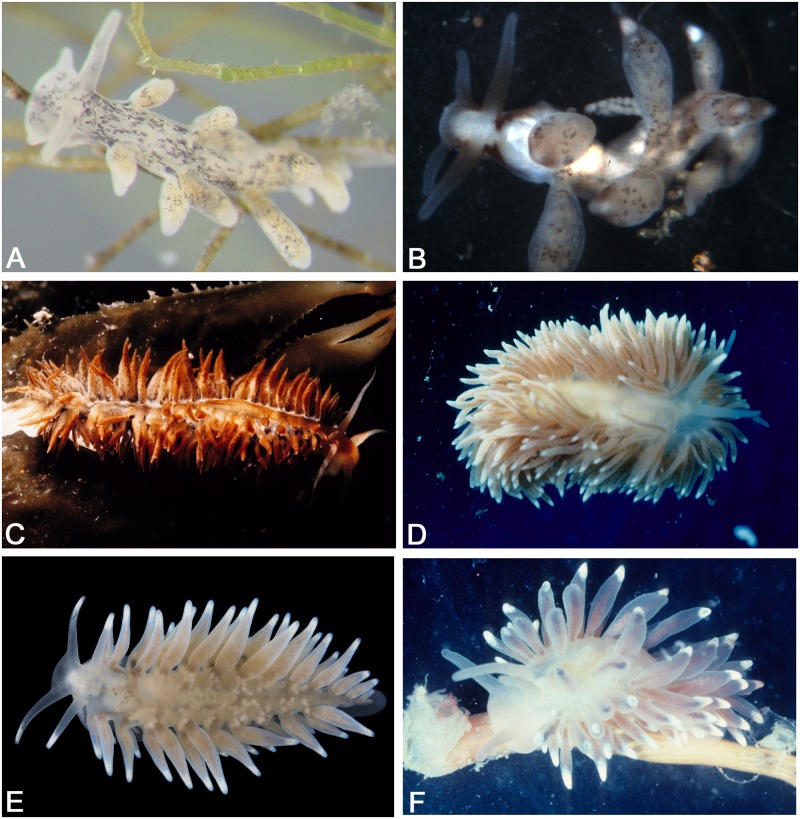
Photographs of the types species of the traditional genera of Fionidae recovered in this study (excluding *Cuthonella* and *Murmania*). (A) *Tenellia adspersa*, photograph by Terrence M. Gosliner. (B) *Tergipes tergipes*, photograph by Terrence M. Gosliner. (C) *Fiona pinnata*, photograph by Terrence M. Gosliner. (D) *Cuthona nana*, photograph by Terrence M. Gosliner. (E) *Calma glaucoides*, photograph by Fredrik Pleijel. (F) *Eubranchus tricolor*, photograph by Terrence M. Gosliner.

*Calma* currently includes only two species, *Calma glaucoides* (Alder and Hancock, 1854) (the type species, Fig 3E) and *Calma gobioophaga* Calado and Urgorri, 2002 (PP = 1, ML = 100). The monophyly of this genus as well as the validity of these two species were recently tested [56]. This small genus is supported by morphological synapomorphies including the lack of anus, intestine, and cnidosacs and the presence of an atypically fused radula [57–59]. These morphological specializations are largely due to their unusual diet of teleost eggs.

### *Cuthona* Alder and Hancock, 1855

Traditionally, *Cuthona* was the largest genus within Tergipedidade (now Fionidae), with more than 80 described species [60]. However, our analyses indicate a completely different scenario. The type species of the genus, *Cuthona nana*, appears nested with the specimens early identified as *C*. *divae* and *C*. *hermitophila*, forming a monophyletic and highly supported clade (PP = 1, ML = 100). The tree topology, the uncorrected *p*-distance as well as the ABGD and PTP analyses indicate that *Cuthona nana*, *C*. *divae* and *C*. *hermitophila* are all conspecific. Traditionally, the first two species have been considered to be closely related since they appear similar externally and are the only species of fionids that have a row of cerata from the posterior cluster that extends anteriorly in front of the anus [15, 61]. They main difference between both species was the presence (*C*. *divae*) or absence (*C*. *nana*) of a bursa copulatrix [14]. However, Brown [15] observed the presence of this structure in the reproductive system of *C*. *nana*, which is also supported by our own research (not shown). Therefore, based this evidence, we suggest that both taxa represent a single species and consider *Cuthona divae* to be a junior synonym of *Cuthona nana*. *Cuthona hermitophila* was recently described by Martynov et al. [62] from the Sea of Japan. He stated that *C*. *hermitophila* is a sibling species to Atlantic *C*. *nana* and Pacific *C*. *divae*, and differs from them both by the presence of a more highly developed central denticle on the rachidian tooth. He also mentioned some minor differences, including the number and position of cerata. Two specimens of *C*. *hermithophila* used for this study were collected in the type locality (Kievka Bay, the Sea of Japan). Each specimen possesses similar rachidian tooth morphology to the original description of this species. However, the differences described by Martynov et al. [62] are likely the result of ontogenetic changes in radular morphology through the late ontogenesis (not shown). Therefore, we consider *C*. *hermitophila*, as a synonym of *C*. *nana*, given that it is nested within the clade of Atlantic specimens of this species.

Additionally, we suggest that *Cratena rubra* Volodchenko, 1941 is likely a junior synonym of *Cuthona nana*. Martynov [10, 63] synonymized *Cratena rubra* with *Flabellina trophina* (Bergh, 1890). Nevertheless, there are several differences between these two species. The radula of *C*. *rubra* is uniseriate [64], whereas the radula of *F*. *trophina* is triseriate [65]. *Cratena rubra* was described with more than 32 rows of cerata [64], but *F*. *trophina* never has so many [65]. Additionally, both species are found in different habitats: silty substrate at 20 m depth for *C*. *rubra*, and rocky walls up to 10 m for *F*. *trophina*. After comparing *Cratena rubra* and *Cuthona nana*, we found similarities in radular morphology, size, number and arrangement of cerata, as well as their habitats. Therefore, we suggest that *Cratena rubra* is not a junior synonym of *Flabellina trophina* but rather of *Cuthona nana*.

Finally, as a monospecific genus, *Cuthona* is characterized by having a row of cerata from the posterior cluster that extends anteriorly in front of the anus. However, the position of *Cuthona* within Fionidae still remains somewhat ambiguous (Figs 1 and 2).

### *Cuthonella* Bergh, 1884

Bergh erected the genus *Cuthonella* [66] for *C*. *abyssicola* Bergh 1884. Also included in this genus *C*. *hiemalis* (Roginskaya, 1987), *C*. *oysoro* (Baba, 1940) and *C*. *soboli* Martynov, 1992 [67]. Traditionally, the main characteristics of this genus are to have the branching of the digestive ducts, with a single row of diverticulae, and a penial gland connected with the vas deferens [3, 22]. Although the validity of this genus has not been questioned as much as other tergipedid taxa (e.g. *Trinchesia*), Millen [68] considered that there were not enough differences between *Cuthona* and *Cuthonella*, and therefore rendered the latter genus as junior synonym of *Cuthona*. However, Martynov [22] resurrected *Cuthonella* for a group of at least six species northern temperate and Arctic tergipedids, including *Cuthonella abyssicola* and *Cuthonella concinna*, characterized by having a “supplementary gland” inserted to the vas deferens and a variable number of branches of the digestive gland. Later he added two more species to this genus–*Cuthonella elenae* Martynov, 2000 and *Cuthonella punicea* [9]. Although material of *C*. *elenae* was not available for this study, our results suggest that *C*. *punicea* belongs to *Tenellia* and is a junior synonym of *T*. *pustulata* (see *Tenellia* Discussion).

Although there was no material of *C*. *abyssicola* available for this study, two other accepted species of *Cuthonella*, *C*. *soboli* and *C*. *hiemalis* were included in the analyses and formed a clade together with *Cuthona concinna* and *C*. *cocoachroma* (PP = 1, ML = 98). The placement of *Cuthona concinna* in *Cuthonella* species agrees with Martynov [22], since he transferred *Cuthona concinna* to *Cuthonella*. In addition, specimens identified as *Cuthonella marisalbi* appeared within the *Cuthona concinna* clade. Martynov [63] stated that *Cuthonella marisalbi* was a junior synonym of *Cuthona concinna*, although later he resurrected *Cuthonella marisalbi* as a valid species [25]. Our molecular results, including the ABGD analyses, support the synonymy proposed by Martynov in 2005 [63], and therefore we render *Cuthonella marisalbi* as a junior synonym of *Cuthona concinna*.

All the species in this genus have brownish-greyish colored cerata. In addition, it should be mentioned that *C*. *cocoachroma* and *C*. *concinna* are similar in external morphology and anatomy, having numerous crowded, elongate cerata arranged in distinct rows, an acleiproctic anus, functional cnidosacs and a typical uniseriate radula [14, 69]. In the original description, the only internal characters that distinguished *C*. *cocoachroma* from *C*. *concinna* are the shape and position of the receptaculum seminis and penial gland, as well as the presence or absence of the bursa copulatrix [14]. Unfortunately, the material examined was not well enough preserved to confirm the differences in the bursa and receptaculum. However, the *p*-distance and the PTP analyses as well as the AGBD method suggest that both species are distinct. *Cuthonella concinna* has more numerous, lighter colored cerata that extend anterior to the rhinophores, while *C*. *cocoachroma*, has darker cerata that are less crowded and do not extend anterior to the rhinophores. Aside from these anatomical differences, the only other difference between these two species is their biogeography: *C*. *cocoachroma* is limited to the eastern Pacific [14] while *C*. *concinna* is a circumpolar species found in both the North Atlantic and northeastern Pacific [14, 70].

Regarding the *Cuthonella soboli* species complex, the external appearance of the specimens that formed the larger clade matches the original description of the species [22]. Additionally, these specimens were collected close to the type locality of Sobol Bay [22]. The two specimens from Kamchatka have some differences in coloration with the true *Cuthonella soboli* (not shown) as well as in radular morphology (not shown), further suggesting that these specimens are part of a species complex and represent an undescribed species of *Cuthonella*.

Here we define *Cuthonella* as fionids with crowded, elongate cerata arranged in distinct rows. The radular tooth has a broad, triangular central cusp and the penial gland connects with the vas deferens.

### *Eubranchus* Forbes, 1838

The Family Eubranchidae has traditionally included 44 species placed in four genera [71]. All of these species have a triseriate radula, a common feature among more primitive aeolids [5] and were originally added to the analyses as an outgroup, since Eubranchidae was hypothesized to be basal to Tergipedidae (now Fionidae). In our phylogenetic study, *Eubranchus* appears nested within Fionidae, being a clade characterized by its triseriate radula and swollen cerata.

Here, two genera and 10 species (including the type species of *Eubranchus*, *E*. *tricolor*, Fig 3F) from the traditional Eubranchidae were part of the dataset. However, our results do not support the validity of *Aenigmastyletus* Martynov, 1998, and therefore, *Eubranchus*, as the older name, takes precedence [50] over *Aenigmastyletus*.

At species level, it was confirmed from a molecular approach the amphiatlantic status of *Eubranchus rupium* as well as its distribution through the Barents Sea, White Sea and Sea of Japan. In addition, *E*. *rupium* appears to be a species complex, since the Californian specimen, as well as the one of specimens from the Sea of Japan, clustered separately. This hidden diversity was also supported by the ABDG and PTP analyses. Despite the very brief original description of *Eubranchus rupium* [72], and because of the type locality (Greenland), we can attribute the largest clade to the true *E*. *rupium*. In addition, the specimen from California matches the original description of *Eubranchus olivaceus* (O'Donoghue, 1922). Several authors considered this species as a junior synonym of *E*. *rupium* [73–74]. Nevertheless, based on our analyses, we confirm the distinctness of *Eubranchus olivaceus*.

Finally, the morphological synapomorphies of *Eubranchus* are swollen cerata and a triseriate radula.

### *Fiona* Alder and Hancock, 1851

*Fiona*, with *F*. *pinnata* (Eschscholtz, 1831) as the type species (Fig 3C), remains the type genus of the family Fionidae in accordance with the ICZN [51], where the type genus of the older family (in this case *Fiona* within the Fionidae) remains the type genus for the revised family. *Fiona*’s relationship to other fionid genera could not be resolved, although Bayesian analyses indicate that it is closely related to the undescribed species originally identified as *Tergipes* sp. (now belonging to a new genus called *Tergiposacca* gen. nov.) (PP = 0.93, ML = 45). The same relationship was found in the Maximum likelihood analysis, but this relationship was weakly supported. This lack of resolution could be due to the fact that H3, which is the most conservative gene of those included here, could not be sequenced for *Fiona pinnata*.

Internal morphology reveals a similar reproductive system to other fionids and like *Tergipes*, the cerata are not clustered in distinct rows and have multiple branches of the digestive gland entering the cerata. *Fiona pinnata* is the only known fionid to possess membranous expansions on the inner margin on the cerata [75–78], which is thought to be an adaptation to pelagic life, spending its life on floating objects and feeding upon cnidarians and barnacles. *Cuthona phoenix* Gosliner, 1981 is also a pelagic fionid, found on floating objects and kelp, where it feeds on hydroids. It has the same branching of the digestive gland and has cerata that are not arranged in rows as in *Tergipes* and *Fiona*. The phylogenetic relationship of *Fiona* and the new genus called *Tergiposacca* gen. nov., as well as the taxonomic position of *Cuthona phoenix*, require additional study.

### *Murmania* Martynov, 2006

*Murmania* is the most recently described genus attributed to the traditional Tergipedidae. This monospecific genus can be identified by a wide body, numerous cerata arranged in rows, a moderately developed notal rim with an elevated ridge, as well as lateral denticles of the radular teeth united in several clusters. Our results support its validity from a molecular approach. In addition, no other fionid species clustered together with *Murmania antiqua*, and therefore this genus remains monotypic.

Finally, this genus appears to be closely related to *Calma* and *Cuthonella*, but this relationship is not supported by the Maximum likelihood analyses (PP = 0.99, ML = 57).

### *Tenellia* A. Costa, 1866

Within Fionidae, analyses recover a clade that includes all members of the genera *Tenellia*, *Trinchesia*, *Phestilla*, *Catriona* and the majority of described and undescribed *Cuthona* species (PP = 0.99, ML = 54). Within this major clade two type species appear, *Tenellia adspersa* and *Trinchesia caerulea*, but not *Cuthona nana*. Since the oldest name is *Tenellia*, and in order to choose the most parsimonious option, we transfer all the species included in this clade over to *Tenellia*, with *T*. *adspersa* as type species (Fig 3A). Transferring all species over to *Tenellia*, however, poses a problem. The original defining character of the genus *Tenellia* is the reduced or lack of oral tentacles, which is not shared by any of the newly transferred species. However, one consistent attribute shared by all members of this lineage is the presence of distinct rows of cerata that are well separated. The discovery of additional morphological synapomorphies requires additional studies and is beyond the scope of this study.

The relationships of species within this clade are also similar to the relationships of clades within Fionidae: strong support values reveal specific clades, but low support values hide their phylogenetic relationships to each other. Further study of more taxa is required to identify other lineages within the *Tenellia* clade.

Detailed study reveals some additional important systematic findings. Specimens previously identified as *Cuthona yamasui* [1, 4] appear to represent two distinct species. Upon examining the original description of this species [79], we concluded that none of the specimens included in this study match with the external coloration depicted by Hamatani [79]. Therefore, both clades belong to two different undescribed species. The true *T*. *yamasui* is depicted in Gosliner et al. [2] as *Cuthona yamasui* (p. 347 top photos) and the two species studied here are shown as *Cuthona* sp. 13 (p. 345 middle photos) and *Cuthona* sp. 14 (p. 345 bottom photos), respectively. A more comprehensive study including the taxon described by Hamatani [79] and a detailed examination of the morphology of these specimens is certainly required in other to clarify this species complex. A similar situation was found inside the *Cuthona speciosa* clade. The specimen CASIZ 176185 clustered separately, but it is sister to the remaining specimens of *C*. *speciosa* included in this study. Based on this tree topology and the *p*-distances (Table 3), these may represent a species complex, but the ABGD analyses did not separate these groups into two species. Photographs of living animals from the larger clade correspond with the original description of the species, but no photographs of the specimen CASIZ 176185 could be found, and therefore we could not determine if there is any external difference between clades. Further studies with more material are needed in order to resolve the identity of the specimen CASIZ 176185.

A third species complex was found within this clade. The two specimens attributed to *Cuthona caerulea* exhibited a high uncorrected *p*-distance for COI (Table 3) and were separated into two different groups by both ABGD and PTP analyses. After examining the original description [80] and the photographs of both specimens, we conclude that the specimen from Ireland is the real *C*. *caerulea*, whereas the Spanish specimen likely represents an undescribed species.

Regarding *C*. *pustulata* and *C*. *punicea*, the former species was originally described by Alder and Hancock [81] from the British Islands. The authors depicted the species as “white, pellucid: branchiae long, linear, obtuse, yellowish orange, granulated with white”, which agrees with the external appearance of all the specimens from this study identified as *C*. *pustulata*. *Cuthona punicea* was described from several specimens from the Canadian Pacific as translucent white or pale peach body with wine-red to purple digestive diverticula [68], matching with the specimen identified as *C*. *punicea* from the Pacific coast of Canada. The two color forms from Maine were observed together and Rudman [82] suggested that *C*. *punicea* is a junior synonym of *C*. *pustulata*. Although material from the type locality of *C*. *pustulata* could not be included in this studied, we consider that both names are referred to the same species, and therefore *C*. *pustulata* would take precedence over *C*. *punicea*. Therefore, the distribution range of *C*. *pustulata* would be very similar to that of *Cuthona nana* (see above). In addition, three specimens of “*C*. *pustulata*” from the Barents Sea clustered together in a separate clade. It seems unlikely they are conspecific with *C*. *pustulata*, and therefore we consider them as representing an undescribed species (*Tenellia* sp. J). Additionally, *Tenellia pustulata* and *Tenellia* sp. J are closely related to *Tenellia fulgens*.

Finally, Rudman [16] considered that the original descriptions of *Phestilla lugubris* and *Phestilla sibogae* referred to the same species, and therefore, stated that the latter was a junior synonym of *P*. *lugubris*. The uncorrected *p*-distance and the ABGD method (not shown) obtained in our study supported this synonymy.

### *Tergipes* Cuvier, 1805

Originally the type genus of Tergipedidae, *Tergipes* now represents fionids with a reduced number of cerata and simple hepatic branching. This genus comprises three or four species [83]: *T*. *antarcticus* Pelseneer, 1903, *T*. *tergipes* (Forsskål in Niebuhr, 1775), *T*. *dicquemari* Risso, 1818 and *T*. *edwardsii* Nordmann, 1844. Two species were sequenced in this study, the type species *Tergipes tergipes* (Fig 3B), and the other an undescribed species from the Indo-Pacific thought to be a species of *Tergipes*. *Tergipes antarcticus* from GenBank was also included in the analyses, however there was a lack of congruence between the Maximum likelihood and Bayesian inference analyses that could be related to a misidentification of the material. In the absence of voucher material, the identity of the species could not be confirmed. Given this ambiguity, the final analyses were conducted excluding this species. Regardless of the inclusion or exclusion of *T*. *antarcticus*, both Bayesian and Maximum likelihood analyses do support the monophyly of this genus for the individuals of *Tergipes tergipes* sampled. It was demonstrated that the Indo-Pacific species is not a species of *Tergipes* and its status is discussed below. These results agree with those obtained by Cámara et al. [84]. Although further analyses, including *T*. *edwardsii*, *T*. *dicquemari* and more material of *T*. *antarcticus* are needed in order to resolve the systematics of this taxon, our study suggests that *Tergipes* is a monospecific genus.

### *Abronica* gen. nov.

LSID urn:lsid:zoobank.org:act:94D8B511-5B28-4C31-9C29-B805C3DE8E9C

**Fig 4:**
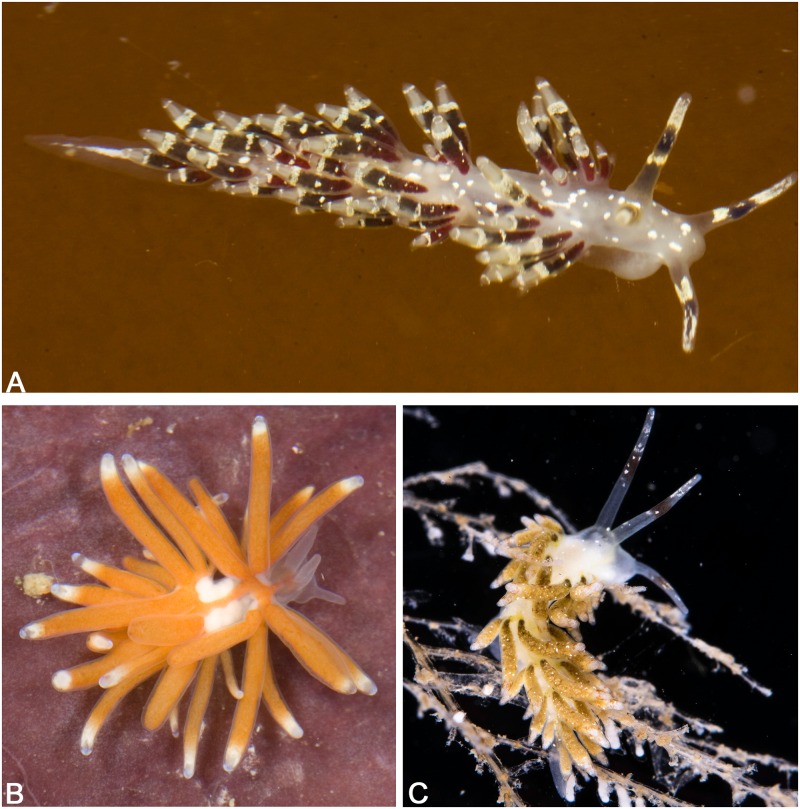
Photographs of the types species of the new genera of Fionidae. (A) *Abronica abronia*, photograph by Terrence M. Gosliner. (B) *Tergiposacca longicerata* photograph by Terrence M. Gosliner. (C) *Rubramoena amoena* photograph by Bernard Picton.

In our analyses, the clade containing *Cuthona abronia* (MacFarland, 1966), *Cuthona purpureoanulata* (Baba, 1961), *Cuthona* sp. 6 and *Cuthona* sp. 7 (PP = 0.99, ML = 94) is consistently distinct from *Cuthona* and all other fionid taxa and is therefore named here as a new genus. The name of *Abronica* refers to its type species by monotypy *Cratena abronia* MacFarland, 1966 (Fig 4A). Purple bands on the rhinophores, opaque white spots on notum and opaque white and/or yellow rings on cerata unite members of this clade. Additionally, both *Cratena abronia* and *Cratena purpureoannulata* Baba, 1961, are distinguished by having an elongate, curved penial stylet ([76, 85] and present study), a feature that has not been observed in other fionids. The presence of this character needs to be verified in the undescribed species tentatively placed in this genus.

### *Rubramoena* gen. nov.

LSID urn:lsid:zoobank.org:act:6A8B49A2-337F-4941-B753-401BD570A16B

All the analyses recovered *Cuthona amoena* and *Cuthona rubescens* in a separate clade (PP = 1, ML = 100 for the concatenate analysis). Both species are apparently very similar, but Picton and Brown [86] stated the main differences between them when they first described *C*. *rubescens*. The etymology of this genus refers to the specific names of both species, being the type species *Rubramoena amoena* (Fig 4C). A translucent white body, dorsum and cerata with whitish-cream speckles, specialized predators upon the hydroid, *Halecium halecinum* (Linnaeus, 1758) and a wide and undulating egg mass are the characteristics shared between these two species of this genus. In addition, both share a similar geographic distribution and can be found in the British Isles [70, 78, 87], the Netherlands [87, 88] and Sweden [89]. *Rubramoena amoena* is also reported from Denmark [87], the Mediterranean coast of France [86] and Spain [90], while *Rubramoena rubescens* can be also found in Norway [91].

Finally, *Rubramoena* gen. nov. might be closely related to the genus *Tergipes*, although this was not supported by the Maximum likelihood analyses (PP = 0.97, ML = 40).

### *Tergiposacca* gen. nov.

LSID urn:lsid:zoobank.org:act:C1EC54AA-9D45-493E-8D34-C5CEB917DAD8

The undescribed species, tentatively attributed to the genus *Tergipes* (*Tergipes* sp. in Gosliner et al. [4]), clusters outside of the larger clade to which *T*. *tergipes* belongs. This outcome is also supported by several anatomical differences that justify the creation of a new genus for this species. *Tergiposacca* gen. nov. combines the saccate form of the egg mass with the genus *Tergipes*. The type species is *T*. *longicerata* n. sp. The new genus is characterized by a translucent, elongate body. Red jaws are visible through the skin. The rhinophores and oral tentacles are smooth and relatively short. The cerata are numerous, long, relatively thick, and of a constant diameter along their length. Some of them have a slight swelling in the upper 1/10. The digestive gland is visible through skin. The cnidosac is translucent with an opaque white ring around the ceratal apex. The reproductive system contains both a receptaculum seminis and a bursa copulatrix. A penial gland is absent and the penis lacks a stylet. The egg mass is white, large, saccate/grape-like.

#### *Tergiposacca longicerata* sp. nov

LSID urn:lsid:zoobank.org:act:9F78E652-C944-44A6-BC89-222F7506F762

*Tergipes* sp.: Gosliner et al. [4], 371.

*Tergipes* sp. 1: Gosliner et al. [2], 356.

*Material examined*: Holotype NMP 041203 (CASIZ Pending number), ST. 20, Aphol’s Rock, Tingloy, Batangas, Philippines, 21 March, 2008, T. Gosliner. Paratypes: CASIZ 203083, 2 specimens, 1 dissected, ST. CAL 48, off Lago de Oro Resort, Calatagan, Batangas, Philippines, 19 May 2014. T. Gosliner. CASIZ 103768, 1 specimen, dissected, CASIZ 208490, 1 specimen with eggs, dissected, ST GAL 43, Shipyard, Puerto Galera, Mindoro Oriental, Philippines, 6 April 2015, T. Gosliner. CASIZ 1777605, 1 specimen, tissue removed for molecular study. ST 4, Aphol’s Rock, Tingloy, Batangas, Philippines, 17 April 2008, T. Gosliner. CASIZ 177442, 1 specimen, ST 12, Arthurs Rock, Mabini, Batangas, Philippines, 19 March, 2008, T. Gosliner. CASIZ 186237, ST HEP 18, Ligpo Point, Bauan, Batangas, Philippines, 2 May, 2011, Alicia Hermosillo. CASIZ 177498, 1 specimen, ST 19, Devil’s Point, Tingloy, Batangas, Philippines, 21 March, 2008, T. Gosliner. CASIZ 085913, 4 specimens, ST 12, Ligpo Island, Bauan, Batangas, Philippines, 24 March, 1993, T. Gosliner. CASIZ 181261, 1 specimen, ST 7, Mainit Bubbles, Mabini, Batangas, Philippines, 16 May 2009, Peri Paleracio. CASIZ 157142, ST. 11, Bethlehem, Tingloy, Batangas, Philippines, 08 May 2001, Ben Castillo.

*Etymology*: The generic name *Tergiposacca* comes them the resemblance of this genus to species of *Tergipes* but this species is distinguished by having saccate egg masses. The species name *longicerata* refers to the elongate cerata that are also characteristic of this species.

*Type locality*: Maricaban Island, Tingloy, Batangas Province, the Philippines.

*Geographical distribution*: This species is known from the western Pacific of Papua New Guinea, the Philippines and southern Japan [4].

*Habitat*: Found commonly under rocks in shallow water (2–5 m).

**Fig 5:**
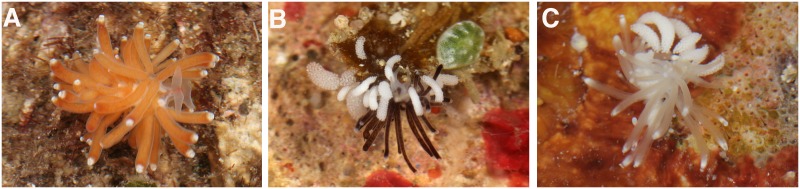
Different morphotypes of *Tergiposacca longicerata* sp. nov. (A) Specimen from Tingloy, the Philippines (CASIZ 177605). (B) Specimen from Puerto Galera, Philippines (CASIZ 208490). (C) Specimen from Tingloy, the Philippines (CASIZ 186236). All the photographs by Terrence M. Gosliner.

*External morphology* (Fig 5): The body is elongate, small and translucent. The foot corners are rounded. Some specimens have orange or burgundy pigmentation over the dorsum. Reddish or orange jaws are visible. The rhinophores and the oral tentacles are translucent and smooth. The rhinophores are longer than the conical oral tentacles.

The cerata are long, cylindrical, with a uniform diameter for most of their length. They extend from the rear of the rhinophores to the tail. The ceratal epithelium is translucent, being visible the ramifications of the digestive gland. The coloration of the digestive gland varies from burgundy to pale grey, including yellow or orange. The upper 1/10 of the cerata there is a subapical white band, and it can be swollen. The cnidosacs are white. The elongate cerata are arranged in three elevated arched clusters on either side of the body. The anteriormost and middle cluster contains 5–12 cerata. The third cluster has 4–10 cerata. The genital aperture is located at the ventral portion of the first ceratal cluster on the right side. The anus is situated on a slightly elevated papilla located within the elevated arch of the second ceratal cluster on the right side.

**Fig 6:**
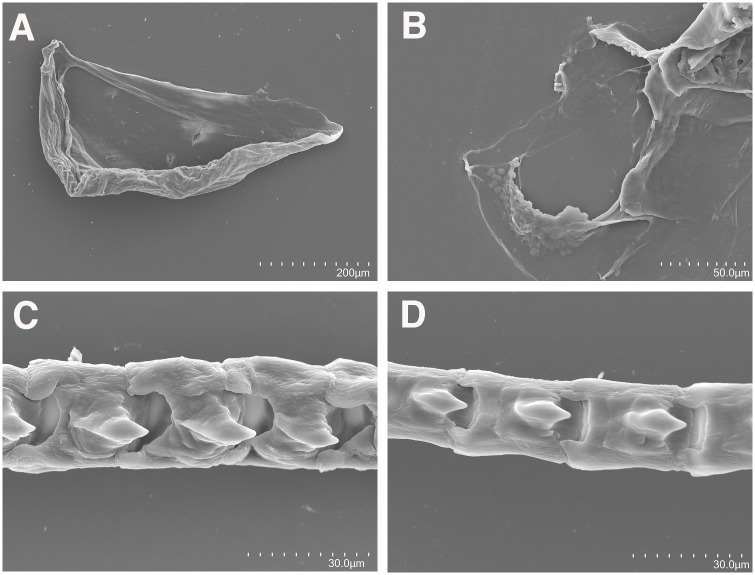
Scanning electron micrographs of the radula, jaws and masticatory edge of *Tergiposacca longicerata* sp. nov. (CASIZ 203083). (A) Jaws of the specimen. (B) Detailed view of the masticatory border. (C-D) Radular teeth.

*Anatomy*: The masticatory border of the jaws is smooth or with a few irregular denticles (Fig 6A and 6B). The radular teeth are very reduced. The radular formula is 21–23 x 0.1.0. The radular teeth have prominent and triangular central cusp, with two denticles on each side (Fig 6C and 6D). The salivary glands are short. Oral glands are absent.

**Fig 7:**
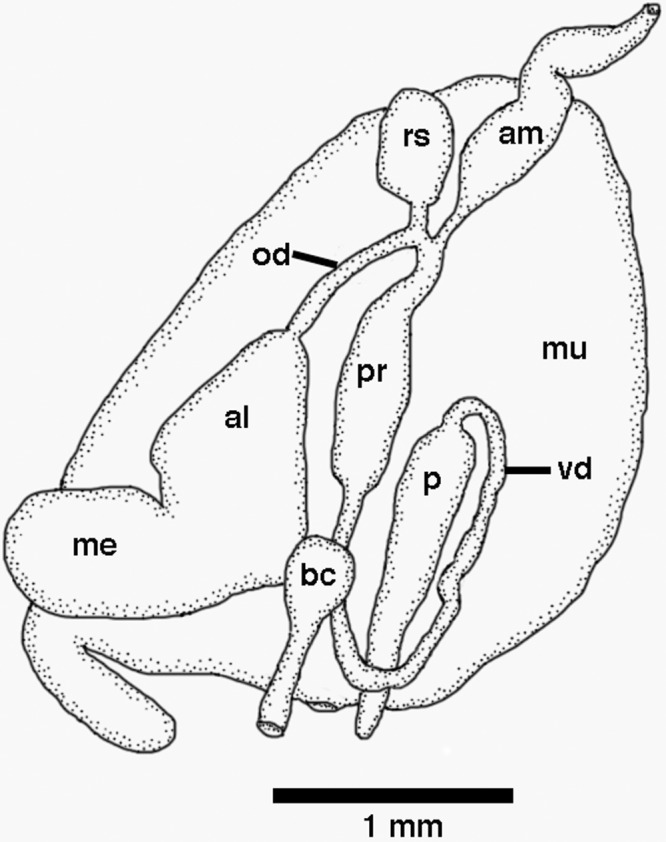
Reproductive system of *Tergiposacca longicerata* sp. nov. (CASIZ 103768). Abbreviations: al, albumen gland; am, ampulla; bc, bursa copulatrix; me, membrane gland; mu, mucous gland; od, oviduct; p, penis; pr, prostate; rs, receptaculum seminis; vd, vas deferens.

The reproductive system is diaulic (Fig 7). The preampullary duct widens into a short ampulla that narrows again before dividing into the oviduct and prostate. The prostate narrows again into a long vas deferens that enters the wider proximal portion of the penial sac. The short oviduct connects to an ovoid receptaculum seminis. The remaining portion of the oviduct departs from the base of the receptaculum and enters the female gland. The female gland mass exits at the female genital aperture, adjacent to the bursa copulatrix, which is large and exits via a relatively long duct. The penis is simple, conical and lacks a penial gland or an apical stylet.

### Hidden diversity

The identification of cryptic or pseudocryptic species by means of traditional taxonomic characters was hampered by the fact that different color forms in nudibranchs have been traditionally attributed to intraspecific variability. This has been particularly true within Aeolidida as demonstrated in the last four years, where more than 20 of these cryptic species were detected and/or described [45, 92–101]. Our study suggests that Fionidae is also rich in species complexes (with four complexes) and previously undetected species diversity. Ignoring cryptic species, leads to an incomplete picture and an underestimation of the real biodiversity. Therefore, future studies are needed in order to determine the identity of these species, to increase the understanding of biodiversity, its distribution and to developing effective conservation strategies that reflect this new knowledge.

### Additional remarks about morphology

Thus far, the main distinguishing morphological characters in the traditional Tergipedidae, here Fionidae, were the radular morphology, the possession of penial stylet and a penial gland [3, 14–15, 20, 23]. Nevertheless, the phylogenetic signal of these characters was highly subjective and depended directly on the opinion of different authors as to the relative importance of different characters. These disagreements have questioned the validity of some fionid genera, especially *Cuthona*, *Trinchesia* and *Catriona*. Employing modern phylogenetic methodology where synapomorphies determine relationships and only clades are recognized rather than paraphyletic lineages has shed tremendous light on these disagreements (e.g., [102]).

The molecular phylogeny presented here however provides us with interesting insights into the evolution of Fionidae. Morphological similarities used to separate traditional taxa appear to be the product of convergence and reflect ecological specializations rather than phylogenetic history, which may explain why the previous classifications based on morphology do not agree with the molecular phylogeny. In order to illustrate these results, Fig 2 maps the evolution of the radular morphology within this family. Recent molecular studies (e.g., [46, 103]) have shown traditional morphology alone fails in many cases. In each instance, we have found new morphological attributes that permit the identification and diagnosis of the lineages illuminated by molecular phylogenetics. Some of the traditional characters, such as the presence of a penial gland situated on the vas deferens rather than at the base of the penis, have been shown to be consistent with the present molecular analysis.

While traditional morphological studies did not establish the most accurate classification of Fionidae, it did help to predict some of its current clades. For instance, *Catriona* is not considered a distinct genus, but the species traditionally classified as *Catriona* do group together in the phylogeny as a clade. This is also the case for the coral-eating species included in *Phestilla*. Previous classification of these taxa based on their dental features may not have been inaccurate, just not completely phylogenetically informative at a “generic level”. Here we have demonstrated that some characters such as such as hepatic branching are diagnostic of the major lineages observed through molecular phylogenetic study. There are likely other underlying morphological similarities uniting these genera that have not been examined in depth, as well as other similarities that have been entirely overlooked. More detailed study of morphology is warranted given the new findings evident in the present phylogenetic study.

## Conclusions

The molecular phylogeny presented here does not agree with any of the traditional classifications of Tergipedidae. Instead of maintaining Tergipedidae as a polyphyletic family, we propose its combination with the traditional families Eubranchidae, Calmidae and Fionidae under the united name of Fionidae and a reclassification of the taxa within the clade. This is proposed based on the molecular analyses in this study, as well as the identification of key morphological characters that are consistent with the strong phylogenetic signal. While this study has clarified the overall phylogenetic structure of Fionidae, this is only a preliminary phylogeny. Hopefully, the work presented here will serve as a starting point for further research into fionid aeolids. The addition of more slowly evolving unlinked markers should help resolve some poorly supported node at the base of the phylogenies presented here. Recently, Churchill et al. [104] included the nuclear 28S gene in their phylogeny of Glaucidae. This provided a good resolution in deeper nodes, and therefore their 28S protocol will be included in further analyses as potentially useful in resolving within-aeolid relationships. In addition, the data set should be completed including specimens of the remaining genus of Fionidae, such as *Myja*, *Subcuthona*, *Leostyletus* and *Guyvalvoria*, as well as other the remaining fionid species not examined in this study. Currently, specimens of *Myja*, *Subcuthona*, and *Guyvalvoria* have not been available for molecular study and most of these taxa have not been observed since their original description. Further morphological investigations are necessary to detect additional anatomical synapomorphies characterizing fionid taxa. Future phylogenetic research is essential to building a more robust phylogeny and clarifying the systematics of Fionidae.

## Supporting Information

S1 Table List of specimens used for phylogenetic analyses. We include both the species names resulting from our morpho-chromatic identification (provisional ids) and the names after analyses (final ids; this only when changes have occurred). Abbreviation: GB, Genbank.
(DOCX)

S1 Fig Molecular phylogeny inferred from partial sequences of the individual genes by Bayesian analysis. (A) H3. (B) COI. (C) 16S (C). Numbers above branches represent posterior probabilities from BI. Numbers below branches indicate bootstrap values for ML. Only nodes supported by BS ≥ 70 and PP ≥ 0.95 are represented.
(TIF)

S2 Fig Molecular phylogeny based on the combined dataset (H3+COI+16S) including *Tergipes antarcticus*. (A) Tree topology from Maximum likelihood analysis; number above branches indicate the bootstrap values. (B) Tree topology from Bayesian inference; numbers above branches represent posterior probabilities. Only nodes supported by BS ≥ 70 and PP ≥ 0.95 are represented.
(TIF)

S3 Fig Molecular phylogeny based on both ML (A) and Bayesian (B) PTP analyses. (TIF)

S4 Fig ABGD analyses for the putative species complex. Trees were extracted from Fig 1. Rectangles represent the groups found by ABGD. (A) “*Eubranchus rupium*”. (B) “*Cuthona yamasui*”. (C) *Cuthona pustulata* and *Cuthona punicea*. (D) *Cuthona concinna*, *Cuthonella marisalbi*, *Cuthonella soboli* and *Cuthona cocoachroma*. Abbreviations: JC, Jukes-Cantor; K, Kimura; and D, Simple distance.
(TIF)

S1 Appendix Extraction, amplification, purification and sequencing protocols followed by each author. (DOCX)
