# Supplementary material for: A Radical Solution: The Phylogeny of the Nudibranch Family Fionidae
Source: PLoS One. 2016 Dec 15;11(12):e0167800. doi: 10.1371/journal.pone.0167800 (PMC5158052; doi:10.1371/journal.pone.0167800)
Supplement: S1 Table — We include both the species names resulting from our morpho-chromatic identification (provisional ids) and the names after analyses (final ids; this only when changes have occurred). Abbreviation: GB, Genbank. (DOCX) [file pone.0167800.s001.docx]

| Family | | Species | | Voucher | Locality | GenBank Accession Nos. | | |
| --- | --- | --- | --- | --- | --- | --- | --- | --- |
| Preliminary ids | Revised ids | Preliminary ids | Revised ids |  |  | H3 | COI | 16S |
| Tritoniidae Lamarck, 1809 |  | *Tritonia pickensi* Marcus and Marcus, 1967 |  | GB |  | HM162549 | HM162717 | HM162642 |
|  |  | *Marionia distincta* Bergh, 1905 |  | GB |  | HM162557 | HM162725 | HM162648 |
| Aeolididae Gray, 1827 |  | *Aeolidia loui* Kienberger, Carmona, Pola, Padula, Gosliner, and Cervera, 2016 |  | CAS173369 | California | KY1284561 | KY128974 | KY128766 |
| Babakinidae Roller, 1973 |  | *Babakina indopacifica* Gosliner,  González-Duarte and Cervera, 2007 |  | GB |  | HM162587 | HM162754 | HM162678 |
| Facelinidae Bergh, 1889 |  | *Cratena pilata* (Gould, 1870) |  | CAS184187 | Massachusetts | KY1284502 | - | KY128709 |
|  |  | *Facelina bostoniensis* (Couthouy, 1838) |  | CAS184184 | New Hampshire | KY1284632 | KY129046 | KY128837 |
|  |  | *Godiva quadricolor* (Barnard, 1927) |  | GB |  | HM162589 | HM162756 | HM162680 |
|  |  | *Phyllodesmium opalescens* Rudman, 1991 |  | GB |  | HQ010449 | HQ010484 | HQ010518 |
|  |  | *Phyllodesmium parangantum* Ortiz and Gosliner, 2003 |  | CAS174440 | Philippines | - | KY129081 | KY128872 |
|  |  | *Phyllodesmium* sp. A* |  | CAS177476 | Philippines | KY1284666 | KY129082 | KY128873 |
|  |  |  |  | CAS181302 | Philippines | KY1284667 | KY129083 | KY128874 |
| Flabellinidae Bergh, 1889 |  | *Flabellina arveloi* Ortea and Espinosa, 1998 |  | CAS179418 | Sao Tome and Principe | KY1284633 | KY129048 | KY128839 |
|  |  |  |  | CAS179419 | Sao Tome and Principe | KY1284634 | KY129049 | KY128840 |
|  |  | *Flabellina bicolor* (Kelaart, 1858) |  | CAS177345 | Philippines | KY1284635 | KY129050 | KY128841 |
|  |  | *Flabellina bilas* (Gosliner and Williams, 1991) |  | CAS177355 | Philippines | KY1284636 | KY129051 | KY128842 |
|  |  | *Flabellina exoptata* Gosliner and Williams, 1991 |  | CAS178322 | Malaysia | KY1284638 | KY129053 | KY128844 |
|  |  | *Flabellina funeka* Gosliner and Griffiths, 1981 |  | CAS176374 | South Africa | KY1284639 | KY129054 | KY128845 |
|  |  | *Flabellina goddardi* Gosliner, 2010 |  | CAS182590 | California | KY1284648 | KY129063 | KY128854 |
|  |  | *Flabellina gracilis* (Alder and Hancock, 1844) |  | CAS183938 | Maine | KY1284640 | KY129055 | KY128846 |
|  |  | *Flabellina iodinea* (J. G. Cooper, 1863) |  | CAS181313a | California | KY1284641 | KY129056 | KY128847 |
|  |  |  |  | CAS181313b | California | KY1284642 | KY129057 | KY128848 |
|  |  |  |  | CAS181313c | California | KY1284643 | KY129058 | KY128849 |
|  |  | *Flabellina macassarana* Bergh, 1905 |  | CAS181283 | Philippines | KY1284644 | KY129059 | KY128850 |
|  |  | *Flabellina pricei* (MacFarland, 1966) |  | CAS114776 | California | KY1284645 | KY129060 | KY128851 |
|  |  | *Flabellina rubrolineata* (O'Donoghue, 1929) |  | CAS177287 | Philippines | KY1284646 | KY129061 | KY128852 |
|  |  | *Flabellina salmonacea* (Couthouy, 1838) |  | CAS183927 | Maine | KY1284647 | KY129062 | KY128853 |
|  |  | *Flabellina trilineata*  (O'Donoghue, 1921) |  | CAS179466 | California | KY1284649 | KY129064 | KY128855 |
|  |  | *Flabellina verrucosa* (M. Sars, 1829) |  | CAS183939 | Maine | KY1284650 | KY129065 | KY128856 |
|  |  | *Flabellina* sp. A |  | CAS181322 | California | KY1284637 | KY129052 | KY128843 |
| Fionidae Gray, 1857 |  | *Fiona pinnata* (Eschscholtz, 1831) |  | CAS179238 | Vanuatu | KY128486 | KY129047 | KY128838 |
|  |  |  |  | GB |  | JX087628 | JX087558 | JX087492 |
| Calmidae Iredale and O'Donoghue, 1923 | Fionidae Gray, 1857 | *Calma glaucoides* (Alder and Hancock, 1854) |  | GNM9030 | Sweden | - | KY128913 | KY128705 |
|  | Fionidae Gray, 1857 |  |  | GNM9091 | Sweden | - | KY128914 | KY128706 |
|  | Fionidae Gray, 1857 | *Calma gobioophaga* Calado and Urgorri, 2002 |  | GB |  | - | HG810896 | HG810890 |
|  | Fionidae Gray, 1857 |  |  | GB |  | - | HG810895 | HG810889 |
| Eubranchidae Odhner, 1934 | Fionidae Gray, 1857 | *Aenigmastyletus alexeii* Martynov, 1998 | *Eubranchus* *alexeii* (Martynov, 1998) | WS3432 | Sea of Japan | KY128487 | KY128900 | KY128692 |
|  | Fionidae Gray, 1857 |  | *Eubranchus* *alexeii* (Martynov, 1998) | WS3433 | Sea of Japan | KY128488 | KY128901 | KY128693 |
|  | Fionidae Gray, 1857 |  | *Eubranchus* *alexeii* (Martynov, 1998) | WS3434 | Sea of Japan | KY128489 | KY128902 | KY128694 |
|  | Fionidae Gray, 1857 | *Eubranchus exiguus* (Alder and Hancock, 1848) |  | WS3456 | Barents Sea | KY1284652 | KY129067 | KY128859 |
|  | Fionidae Gray, 1857 |  |  | GNM9092 | Scotland | KY1284615 | KY129029 | KY128820 |
|  | Fionidae Gray, 1857 | *Eubranchus farrani* (Alder and Hancock, 1844) |  | GB |  | - | - | AJ223396 |
|  | Fionidae Gray, 1857 |  |  | GNM9093 | Sweden | KY1284614 | KY129028 | KY128819 |
|  | Fionidae Gray, 1857 | *Eubranchus mandapamensis* (Rao, 1968) |  | CAS177750a | Philippines | KY1284621 | KY129035 | KY128826 |
|  | Fionidae Gray, 1857 |  |  | CAS177750b | Philippines | KY1284622 | KY129036 | KY128827 |
|  | Fionidae Gray, 1857 | *Eubranchus odhneri* (Derjugin & Gurjanova, 1926) |  | WS3435 | White Sea | KY128490 | KY128903 | KY128695 |
|  | Fionidae Gray, 1857 | *Eubranchus pallidus* (Alder & Hancock, 1842) |  | WS3454 | Barents Sea | KY1284619 | KY129033 | KY128824 |
|  | Fionidae Gray, 1857 |  |  | GNM9094 | Scotland | KY1284616 | KY129030 | KY128821 |
|  | Fionidae Gray, 1857 | *Eubranchus rupium*  (Møller, 1842) | *Eubranchus* *olivaceus* (O'Donoghue, 1922) | CAS181133 | California | KY1284623 | KY129037 | KY128828 |
|  | Fionidae Gray, 1857 |  |  | CAS183925 | Maine | KY1284620 | KY129034 | KY128825 |
|  | Fionidae Gray, 1857 |  |  | CAS183929 | Maine | KY1284630 | KY129044 | KY128835 |
|  | Fionidae Gray, 1857 |  |  | WS3457 | White Sea | KY1284653 | KY129068 | KY128860 |
|  | Fionidae Gray, 1857 |  |  | WS3458 | White Sea | KY1284654 | KY129069 | KY128861 |
|  | Fionidae Gray, 1857 |  |  | WS3459 | White Sea | KY1284655 | KY129070 | KY128862 |
|  | Fionidae Gray, 1857 |  |  | WS3460 | Barents Sea | KY1284656 | KY129071 | KY128858 |
|  | Fionidae Gray, 1857 |  |  | WS3461 | Barents Sea | KY1284657 | KY129072 | KY128863 |
|  | Fionidae Gray, 1857 |  |  | WS3462 | Sea of Japan | KY1284658 | KY129073 | KY128864 |
|  | Fionidae Gray, 1857 |  |  | GNM9095 | Sweden | KY1284617 | KY129031 | KY128822 |
|  | Fionidae Gray, 1857 |  |  | AC17-20 | Sea of Japan | KY1284624 | KY129038 | KY128829 |
|  | Fionidae Gray, 1857 |  | *Eubranchus* sp. A | AC17-21 | Sea of Japan | KY1284625 | KY129039 | KY128830 |
|  | Fionidae Gray, 1857 | *Eubranchus tricolor Forbes, 1838* |  | WS3478 | Barents Sea | KY1284631 |  | KY128836 |
|  | Fionidae Gray, 1857 |  |  | GNM9096 | Sweden | KY1284618 | KY129032 | KY128823 |
|  | Fionidae Gray, 1857 | *Eubranchus vittatus* (Alder & Hancock, 1842) |  | GNM9097 | Great Britain | - | KY129045 | - |
|  | Fionidae Gray, 1857 | *Eubranchus* sp. 3 |  | CAS181292a | Philippines | KY1284626 | KY129040 | KY128831 |
|  | Fionidae Gray, 1857 |  |  | CAS181292b | Philippines | KY1284627 | KY129041 | KY128832 |
|  | Fionidae Gray, 1857 |  |  | CAS181292c | Philippines | KY1284628 | KY129042 | KY128833 |
|  | Fionidae Gray, 1857 |  |  | CAS181292d | Philippines | KY1284629 | KY129043 | KY128834 |
| Tergipedidae Bergh, 1889 | Fionidae Gray, 1857 | *Catriona columbiana* (O'Donoghue, 1922) | *Tenellia columbiana*  (O'Donoghue, 1922) | CAS185195 | California | KY128493 | KY128906 | KY128698 |
|  | Fionidae Gray, 1857 | *Catriona gymnota* (Couthouy, 1838) | *Tenellia gymnota*  (Couthouy, 1838) | CAS184182 | New Hampshire | KY128494 | KY128907 | KY128699 |
|  | Fionidae Gray, 1857 |  | *Tenellia gymnota*  (Couthouy, 1838) | CAS184188 | New Hampshire | KY128495 | KY128908 | KY128700 |
|  | Fionidae Gray, 1857 |  | *Tenellia gymnota*  (Couthouy, 1838) | GNM8948 | Väderö Islands, Sweden | KY1284500 | - | KY128707 |
|  | Fionidae Gray, 1857 | *Catriona* cf *maua* Ev. Marcus and Er. Marcus, 1960 | *Tenellia* cf *maua* (Ev. Marcus and Er. Marcus, 1960) | CAS179403 | Sao Tome and Principe | KY128492 | KY128905 | KY128697 |
|  | Fionidae Gray, 1857 | *Catriona* sp. A | *Tenellia* sp. D | CAS185133 | Hawaii | KY128496 | KY128909 | KY128701 |
|  | Fionidae Gray, 1857 |  | *Tenellia* sp. D | CAS185139 | Hawaii | KY128497 | KY128910 | KY128702 |
|  | Fionidae Gray, 1857 | *Cuthona abronia*  (MacFarland, 1966) | *Abronica abronia*  (MacFarland, 1966) | CAS174485 | California | KY1284504 | KY128917 | KY128712 |
|  | Fionidae Gray, 1857 |  | *Abronica abronia*  (MacFarland, 1966) | CAS179463a | California | KY1284505 | KY128918 | KY128713 |
|  | Fionidae Gray, 1857 |  | *Abronica abronia*  (MacFarland, 1966) | CAS179463b | California | KY1284506 | KY128919 | KY128714 |
|  | Fionidae Gray, 1857 |  | *Abronica abronia*  (MacFarland, 1966) | CAS179463c | California | KY1284507 | KY128920 | KY128715 |
|  | Fionidae Gray, 1857 |  | *Abronica abronia*  (MacFarland, 1966) | CAS181319 | California | KY1284508 | KY128919 | KY128716 |
|  | Fionidae Gray, 1857 | *Cuthona albocrusta* (MacFarland, 1966) | *Tenellia albocrusta* (MacFarland, 1966) | CAS181520 | Canada | KY1284509 | - | - |
|  | Fionidae Gray, 1857 | *Cuthona amoena* (Alder & Hancock, 1845) | *Rubramoena*  *amoena* (Alder & Hancock, 1845) | GNM9098 | Great Britain | KY128491 | KY128904 | KY12869696 |
|  | Fionidae Gray, 1857 | *Cuthona caerulea* (Montagu, 1804) | *Tenellia* sp. H | CAS185199 | Spain | KY1284510 | KY128922 | KY128717 |
|  | Fionidae Gray, 1857 |  | *Tenellia* sp. I | GNM9099 | Great Britain | KY128498 | KY128911 | KY128703 |
|  | Fionidae Gray, 1857 | *Cuthona cocoachroma* Williams and Gosliner, 1979 | *Cuthonella cocoachroma* (Williams and Gosliner, 1979) | CAS181307b | California | KY1284515 | KY128927 | KY128722 |
|  | Fionidae Gray, 1857 |  | *Cuthonella cocoachroma* (Williams and Gosliner, 1979) | CAS181307c | California | KY1284516 | KY128928 | KY128723 |
|  | Fionidae Gray, 1857 |  | *Cuthonella cocoachroma* (Williams and Gosliner, 1979) | CAS181307e | California | KY1284518 | KY128930 | KY128725 |
|  | Fionidae Gray, 1857 |  | *Cuthonella cocoachroma* (Williams and Gosliner, 1979) | CAS179471 | California | KY1284513 | KY128925 | KY128720 |
|  | Fionidae Gray, 1857 |  | *Cuthonella cocoachroma* (Williams and Gosliner, 1979) | CAS185193 | California | KY1284519 | KY128931 | KY128726 |
|  | Fionidae Gray, 1857 | *Cuthona concinna* (Alder and Hancock, 1843) | *Cuthonella concinna* (Alder and Hancock, 1843) | CAS182701 | New Hampshire | KY1284521 | KY128933 | KY128728 |
|  | Fionidae Gray, 1857 |  | *Cuthonella concinna* (Alder and Hancock, 1843) | CAS182702 | Maine | KY1284522 | KY128934 | KY128729 |
|  | Fionidae Gray, 1857 |  | *Cuthonella concinna* (Alder and Hancock, 1843) | CAS183926 | Maine | KY1284523 | KY128935 | KY128730 |
|  | Fionidae Gray, 1857 |  | *Cuthonella concinna* (Alder and Hancock, 1843) | CAS183932 | Maine | KY1284524 | KY128936 | KY128731 |
|  | Fionidae Gray, 1857 |  | *Cuthonella concinna* (Alder and Hancock, 1843) | CAS181522b | Alaska | KY1284520 | KY128932 | KY128727 |
|  | Fionidae Gray, 1857 |  | *Cuthonella concinna* (Alder and Hancock, 1843) | CAS179469 | California | KY1284512 | KY128924 | KY128719 |
|  | Fionidae Gray, 1857 |  | *Cuthonella concinna* (Alder and Hancock, 1843) | CAS181307a | California | KY1284514 | KY128926 | KY128721 |
|  | Fionidae Gray, 1857 |  | *Cuthonella concinna* (Alder and Hancock, 1843) | CAS181307d | California | KY1284517 | KY128929 | KY128724 |
|  | Fionidae Gray, 1857 | *Cuthona divae* (Er. Marcus, 1961) | *Cuthona nana* (Alder and Hancock, 1842) | CAS174478 | California | KY1284525 | KY128937 | KY128732 |
|  | Fionidae Gray, 1857 |  | *Cuthona nana* (Alder and Hancock, 1842) | CAS174482 | California | KY1284526 | KY128938 | KY128733 |
|  | Fionidae Gray, 1857 |  | *Cuthona nana* (Alder and Hancock, 1842) | CAS174495 | California | KY1284527 | KY128939 | KY128734 |
|  | Fionidae Gray, 1857 |  | *Cuthona nana* (Alder and Hancock, 1842) | CAS179464a | California | KY1284528 | KY128940 | KY128735 |
|  | Fionidae Gray, 1857 |  | *Cuthona nana* (Alder and Hancock, 1842) | CAS179464b | California | KY1284529 | KY128941 | KY128736 |
|  | Fionidae Gray, 1857 |  | *Cuthona nana* (Alder and Hancock, 1842) | CAS179470a | California | KY1284530 | KY128942 | KY128737 |
|  | Fionidae Gray, 1857 |  | *Cuthona nana* (Alder and Hancock, 1842) | CAS179470b | California | KY1284531 | KY128943 | KY128738 |
|  | Fionidae Gray, 1857 |  | *Cuthona nana* (Alder and Hancock, 1842) | CAS179477 | California | KY1284532 | KY128944 | KY128739 |
|  | Fionidae Gray, 1857 |  | *Cuthona nana* (Alder and Hancock, 1842) | CAS179483 | California | KY1284533 | KY128945 | KY128740 |
|  | Fionidae Gray, 1857 |  | *Cuthona nana* (Alder and Hancock, 1842) | CAS181316 | California | KY1284534 | KY128946 | KY128741 |
|  | Fionidae Gray, 1857 | *Cuthona flavovulta* (MacFarland, 1966) | *Tenellia flavovulta* (MacFarland, 1966) | CAS179468 | California | KY1284536 | KY128948 | KY128743 |
|  | Fionidae Gray, 1857 |  | *Tenellia flavovulta* (MacFarland, 1966) | CAS174479 | California | KY1284535 | KY128947 | KY128742 |
|  | Fionidae Gray, 1857 |  | *Tenellia flavovulta* (MacFarland, 1966) | CAS179484 | California | KY1284537 | KY128949 | KY128744 |
|  | Fionidae Gray, 1857 |  | *Tenellia flavovulta* (MacFarland, 1966) | CAS181132 | California | KY1284538 | KY128950 | KY128745 |
|  | Fionidae Gray, 1857 | *Cuthona foliata* (Forbes & Goodsir, 1839) | *Tenellia foliata* (Forbes & Goodsir, 1839) | GNM9100 | Ireland | KY128499 | KY128912 | KY128704 |
|  | Fionidae Gray, 1857 | *Cuthona fulgens* (MacFarland, 1966) | *Tenellia fulgens* (MacFarland, 1966) | CAS174484 | California | KY1284539 | KY128951 | KY128746 |
|  | Fionidae Gray, 1857 |  | *Tenellia fulgens* (MacFarland, 1966) | CAS185194 | California | KY1284540 | KY128952 | KY128747 |
|  | Fionidae Gray, 1857 | *Cuthona hermitophila* Martynov, Sanamyan, Korshunova, 2015 | *Cuthona nana* (Alder and Hancock, 1842) | AC19-29 | Sea of Japan | KY1284541 | KY128953 | KY128711 |
|  | Fionidae Gray, 1857 |  | *Cuthona nana* (Alder and Hancock, 1842) | AC29-52 | Sea of Japan | - | KY128954 | - |
|  | Fionidae Gray, 1857 | *Cuthona lagunae* (O'Donoghue, 1926) | *Tenellia lagunae*  (O'Donoghue, 1926) | CAS175583 | California | KY1284542 | KY128955 | KY128748 |
|  | Fionidae Gray, 1857 |  | *Tenellia lagunae*  (O'Donoghue, 1926) | CAS179465a | California | KY1284543 | KY128956 | KY128749 |
|  | Fionidae Gray, 1857 |  | *Tenellia lagunae*  (O'Donoghue, 1926) | CAS179465b | California | KY1284544 | KY128957 | KY128750 |
|  | Fionidae Gray, 1857 |  | *Tenellia lagunae* (O'Donoghue, 1926) | CAS179465c | California | KY1284545 | KY128958 | KY128751 |
|  | Fionidae Gray, 1857 |  | *Tenellia lagunae* (O'Donoghue, 1926) | CAS179465d | California | KY1284546 | KY128959 | KY128752 |
|  | Fionidae Gray, 1857 |  | *Tenellia lagunae* (O'Donoghue, 1926) | CAS179465e | California | KY1284547 | KY128960 | KY128753 |
|  | Fionidae Gray, 1857 | *Cuthona nana* (Alder and Hancock, 1842) |  | CAS182700 | New Hampshire | KY1284548 | KY128961 | KY128754 |
|  | Fionidae Gray, 1857 |  |  | WS3436 | Sea of Japan | KY1284550 | KY128963 | - |
|  | Fionidae Gray, 1857 |  |  | WS3437 | Barents Sea | KY1284552 | KY128965 | KY128757 |
|  | Fionidae Gray, 1857 |  |  | GNM9101 | Sweden | KY1284501 | KY128915 | KY128708 |
|  | Fionidae Gray, 1857 |  |  | AC14-10 | Sea of Japan | KY1284549 | KY128962 | KY128755 |
|  | Fionidae Gray, 1857 |  |  | AC5-2 | Sea of Japan | - | KY128966 | - |
|  | Fionidae Gray, 1857 |  |  | AC22-14 | Netherlands | KY1284551 | KY128964 | KY128756 |
|  | Fionidae Gray, 1857 | *Cuthona ornata* Baba, 1937 | *Tenellia ornata* Baba, 1937 | CAS180344 | Hawaii | KY1284553 | KY128967 | KY128758 |
|  | Fionidae Gray, 1857 | *Cuthona poritophages* Rudman, 1979 | *Tenellia poritophages* Rudman, 1979 | CAS177737 | Philippines | KY1284554 | KY128968 | KY128759 |
|  | Fionidae Gray, 1857 |  | *Tenellia poritophages* Rudman, 1979 | CAS177738 | Philippines | KY1284555 | KY128969 | KY128760 |
|  | Fionidae Gray, 1857 | *Cuthona punicea* Millen, 1986 | *Tenellia* cf *pustulata* (Alder and Hancock, 1854) | CAS181525 | Canada | KY1284556 | - | KY128761 |
|  | Fionidae Gray, 1857 | *Cuthona purpureoanulata* (Baba, 1961) | *Abronica purpureoanulata* (Baba, 1961) | CAS177607 | Philippines | KY1284557 | KY128970 | KY128762 |
|  | Fionidae Gray, 1857 |  | *Abronica purpureoanulata* (Baba, 1961) | CAS181296 | Philippines | KY1284558 | KY128971 | KY128763 |
|  | Fionidae Gray, 1857 | *Cuthona pustulata* (Alder and Hancock, 1854) | *Tenellia* cf *pustulata* (Alder and Hancock, 1854) | CAS183930 | Maine | KY1284559 | KY128972 | KY128764 |
|  | Fionidae Gray, 1857 |  | *Tenellia* cf *pustulata* (Alder and Hancock, 1854) | CAS183933 | Maine | KY1284560 | KY128973 | KY128765 |
|  | Fionidae Gray, 1857 |  | *Tenellia* cf *pustulata* (Alder and Hancock, 1854) | WS3466 | White sea | KY1284677 | KY129094 | KY128885 |
|  | Fionidae Gray, 1857 |  | *Tenellia* cf *pustulata* (Alder and Hancock, 1854) | WS3467 | Barents sea | KY1284678 | KY129095 | KY128886 |
|  | Fionidae Gray, 1857 |  | *Tenellia* cf *pustulata* (Alder and Hancock, 1854) | WS3468 | Barents sea | KY1284679 | KY129096 | KY128887 |
|  | Fionidae Gray, 1857 |  | *Tenellia* cf *pustulata* (Alder and Hancock, 1854) | WS3469 | Barents sea | KY1284680 | KY129097 | KY128888 |
|  | Fionidae Gray, 1857 |  | *Tenellia* cf *pustulata* (Alder and Hancock, 1854) | WS3470 | Barents sea | KY1284681 | KY129098 | KY128889 |
|  | Fionidae Gray, 1857 |  | *Tenellia* cf *pustulata* (Alder and Hancock, 1854) | WS3471 | Barents sea | KY1284682 | KY129099 | KY128890 |
|  | Fionidae Gray, 1857 |  | *Tenellia* cf *pustulata* (Alder and Hancock, 1854) | WS3472 | Barents sea | KY1284683 | KY129100 | KY128891 |
|  | Fionidae Gray, 1857 |  | *Tenellia* sp. J | WS3473 | Barents sea | KY1284684 | KY129101 | KY128892 |
|  | Fionidae Gray, 1857 |  | *Tenellia* sp. J | WS3474 | Barents sea | KY1284685 | KY129102 | KY128893 |
|  | Fionidae Gray, 1857 |  | *Tenellia* sp. J | WS3475 | Barents sea | KY1284686 | KY129103 | KY128894 |
|  | Fionidae Gray, 1857 |  | *Tenellia* cf *pustulata* (Alder and Hancock, 1854) | WS2003 | White Sea | KY1284687 | - | KY128895 |
|  | Fionidae Gray, 1857 |  | *Tenellia* cf *pustulata* (Alder and Hancock, 1854) | WS2133 | White Sea | KY1284688 | - | KY128896 |
|  | Fionidae Gray, 1857 | *Cuthona rubescens* Picton & Brown, 1978 | *Rubramoena*  *rubescens* (Picton & Brown, 1978) | GNM9102 | Great Britain | KY1284503 | KY128916 | KY128710 |
|  | Fionidae Gray, 1857 | *Cuthona sibogae* (Bergh, 1905) | *Tenellia sibogae* (Bergh, 1905) | CAS177489 | Philippines | KY1284562 | KY128975 | KY128767 |
|  | Fionidae Gray, 1857 | *Cuthona speciosa* (Macnae, 1954) | *Tenellia* sp. M | CAS176185 | South Africa | KY1284582 | KY128995 | KY128787 |
|  | Fionidae Gray, 1857 |  | *Tenellia speciosa* (Macnae, 1954) | CAS176913 | South Africa | KY1284583 | KY128996 | KY128788 |
|  | Fionidae Gray, 1857 |  | *Tenellia speciosa* (Macnae, 1954) | CAS176914 | South Africa | KY1284584 | KY128997 | KY128789 |
|  | Fionidae Gray, 1857 |  | *Tenellia speciosa* (Macnae, 1954) | CAS176954 | South Africa | KY1284585 | KY128998 | KY128790 |
|  | Fionidae Gray, 1857 | *Cuthona viridis* (Forbes, 1840) | *Tenellia viridis* (Forbes, 1840) | WS3476 | White Sea | KY1284689 | KY129104 | KY128897 |
|  | Fionidae Gray, 1857 |  | *Tenellia viridis* (Forbes, 1840) | WS3556 |  | KY1284691 | - | KY128899 |
|  | Fionidae Gray, 1857 |  | *Tenellia viridis* (Forbes, 1840) | WS3477 | Barents Sea | KY1284690 | KY129105 | KY128898 |
|  | Fionidae Gray, 1857 |  | *Tenellia viridis* (Forbes, 1840) | GNM9103 | Great Britain | KY1284613 | KY129027 | KY128818 |
|  | Fionidae Gray, 1857 | *Cuthona yamasui* Hamatani, 1993 | *Tenellia* sp. E | CAS176737 | Malaysia | KY1284586 | KY128999 | KY128791 |
|  | Fionidae Gray, 1857 |  | *Tenellia* sp. E | CAS176739a | Malaysia | KY1284587 | KY129000 | KY128792 |
|  | Fionidae Gray, 1857 |  | *Tenellia* sp. E | CAS176739b | Malaysia | KY1284588 | KY129001 | KY128793 |
|  | Fionidae Gray, 1857 |  | *Tenellia* sp. E | CAS181298 | Philippines | KY1284593 | KY129006 | KY128798 |
|  | Fionidae Gray, 1857 |  | *Tenellia* sp. E | CAS177552 | Philippines | KY1284590 | KY129003 | KY128795 |
|  | Fionidae Gray, 1857 |  | *Tenellia* sp. F | CAS177553 | Philippines | KY1284591 | KY129004 | KY128796 |
|  | Fionidae Gray, 1857 |  | *Tenellia* sp. F | CAS177554 | Philippines | KY1284592 | KY129005 | KY128797 |
|  | Fionidae Gray, 1857 |  | *Tenellia* sp. F | CAS177469 | Philippines | KY1284589 | KY129002 | KY128794 |
|  | Fionidae Gray, 1857 | *Cuthona* sp. 2 | *Tenellia* sp. 2 | CAS177293 | Philippines | KY1284572 | KY128985 | KY128777 |
|  | Fionidae Gray, 1857 |  | *Tenellia* sp. 2 | CAS177389 | Philippines | KY1284573 | KY128986 | KY128778 |
|  | Fionidae Gray, 1857 |  | *Tenellia* sp. 2 | CAS178436 | Line Islands | KY1284574 | KY128987 | KY128779 |
|  | Fionidae Gray, 1857 | *Cuthona* sp. 6 | *Abronica* sp. 6 | CAS177350 | Philippines | KY1284575 | KY128988 | KY128780 |
|  | Fionidae Gray, 1857 |  | *Abronica* sp. 6 | CAS177353 | Philippines | KY1284576 | KY128989 | KY128781 |
|  | Fionidae Gray, 1857 |  | *Abronica* sp. 6 | CAS177417 | Philippines | KY1284577 | KY128990 | KY128782 |
|  | Fionidae Gray, 1857 | *Cuthona* sp. 7 | *Abronica* sp. 7 | CAS179946 | Hawaii | KY1284578 | KY128991 | KY128783 |
|  | Fionidae Gray, 1857 | *Cuthona* sp. 10 | *Tenellia* sp. 10 | CAS176732 | Malaysia | KY1284563 | KY128976 | KY128768 |
|  | Fionidae Gray, 1857 |  | *Tenellia* sp. 10 | CAS177583 | Philippines | KY1284564 | KY128977 | KY128769 |
|  | Fionidae Gray, 1857 | *Cuthona* sp. 12 | *Tenellia* sp. 12 | CAS176733 | Malaysia | KY1284565 | KY128978 | KY128770 |
|  | Fionidae Gray, 1857 | *Cuthona* sp. 15 | *Tenellia* sp. 15 | CAS181254 | Philippines | KY1284567 | KY128980 | KY128772 |
|  | Fionidae Gray, 1857 | *Cuthona* sp. 17 | *Tenellia* sp. 17 | CAS177725 | Philippines | KY1284568 | KY128981 | KY128773 |
|  | Fionidae Gray, 1857 | *Cuthona* sp. 19 | *Tenellia* sp. 19 | CAS177316 | Philippines | KY1284569 | KY128982 | KY128774 |
|  | Fionidae Gray, 1857 |  | *Tenellia* sp. K | CAS177722 | Philippines | KY1284570 | KY128983 | KY128775 |
|  | Fionidae Gray, 1857 | *Cuthona* sp. 29 | *Tenellia* sp. 29 | CAS180395 | Philippines | KY1284571 | KY128984 | KY128776 |
|  | Fionidae Gray, 1857 | *Cuthona* sp. A | *Tenellia* sp. A | CAS177747 | Philippines | KY1284579 | KY128992 | KY128784 |
|  | Fionidae Gray, 1857 | *Cuthona* sp. B | *Tenellia* sp. B | CAS180404 | Philippines | KY1284580 | KY128993 | KY128785 |
|  | Fionidae Gray, 1857 |  | *Tenellia* sp. B | CAS181244 | Philippines | KY1284581 | KY128994 | KY128786 |
|  | Fionidae Gray, 1857 | *Cuthona* sp. C | *Tenellia* sp. C | CAS176952 | South Africa | KY1284511 | KY128923 | KY128718 |
|  | Fionidae Gray, 1857 | *Cuthona* sp. D** | *Tenellia* sp. G | CAS176796 | Hawaii | KY1284566 | KY128979 | KY128771 |
|  | Fionidae Gray, 1857 | *Cuthonella hiemalis* (Roginskaya, 1987) |  | WS3438 | White Sea | KY1284595 | KY129007 | KY128799 |
|  | Fionidae Gray, 1857 |  |  | WS3439 | White Sea | KY1284596 | KY129008 | KY128800 |
|  | Fionidae Gray, 1857 |  |  | WS3440 | White Sea | KY1284597 | KY129009 | KY128801 |
|  | Fionidae Gray, 1857 |  |  | WS3441 | White Sea | KY1284598 | KY129010 | KY128802 |
|  | Fionidae Gray, 1857 |  |  | WS3442 | White Sea | KY1284599 | KY129011 | KY128803 |
|  | Fionidae Gray, 1857 | *Cuthonella marisalbi* (Roginskaya, 1963) | *Cuthonella concinna* (Alder and Hancock, 1843) | WS3443 | White Sea | KY1284600 | KY129012 | KY128804 |
|  | Fionidae Gray, 1857 |  | *Cuthonella concinna* (Alder and Hancock, 1843) | WS3444 | Barents Sea | KY1284611 | KY129013 | KY128817 |
|  | Fionidae Gray, 1857 |  | *Cuthonella concinna* (Alder and Hancock, 1843) | WS3445 | Barents Sea | KY1284612 | KY129014 | KY128805 |
|  | Fionidae Gray, 1857 |  | *Cuthonella concinna* (Alder and Hancock, 1843) | WS3446 | White Sea | KY1284601 | KY129015 | KY128806 |
|  | Fionidae Gray, 1857 |  | *Cuthonella concinna* (Alder and Hancock, 1843) | WS3447 | White Sea | KY1284602 | KY129016 | KY128807 |
|  | Fionidae Gray, 1857 |  | *Cuthonella concinna* (Alder and Hancock, 1843) | WS3448 | White Sea | - | KY129017 | - |
|  | Fionidae Gray, 1857 |  | *Cuthonella concinna* (Alder and Hancock, 1843) | WS3449 | White Sea | KY1284603 | KY129018 | KY128808 |
|  | Fionidae Gray, 1857 |  | *Cuthonella concinna* (Alder and Hancock, 1843) | WS2893 | White Sea | KY1284604 | - | KY128809 |
|  | Fionidae Gray, 1857 | *Cuthonella soboli* Martynov, 1992 | *Cuthonella* sp. A | WS3450 | NW Pacific, Kamchatka | KY1284594 | KY129019 | KY128810 |
|  |  |  | *Cuthonella* sp. A | WS3452 | NW Pacific, Kamchatka | KY1284608 | KY129023 | KY128814 |
|  | Fionidae Gray, 1857 |  |  | WS3451 | Sea of Japan | KY1284606 | KY129021 | KY128812 |
|  | Fionidae Gray, 1857 |  |  | WS3453 | Sea of Japan | KY1284609 | KY129024 | KY128815 |
|  | Fionidae Gray, 1857 |  |  | AC1-4 | Sea of Japan | KY1284605 | KY129020 | KY128811 |
|  | Fionidae Gray, 1857 |  |  | AC20-11 | Sea of Japan | KY1284607 | KY129022 | KY128813 |
|  | Fionidae Gray, 1857 |  |  | AC5-9 | Sea of Japan | KY1284610 | KY129026 | KY128816 |
|  | Fionidae Gray, 1857 |  |  | AC19-33 | Sea of Japan | - | KY129025 | - |
|  | Fionidae Gray, 1857 | *Murmania antiqua* Martynov, 2006 |  | WS3455 | Kara Sea | KY1284651 | KY129066 | KY128857 |
|  | Fionidae Gray, 1857 | *Phestilla lugubris* (Bergh, 1870) | *Tenellia lugubris* (Bergh, 1870) | CAS177437 | Philippines | KY1284660 | KY129075 | KY128866 |
|  | Fionidae Gray, 1857 | *Phestilla melanobrachia* Bergh, 1874 | *Tenellia melanobrachia* (Bergh, 1874) | CAS167974a | Papua New Guinea | KY1284661 | KY129076 | KY128867 |
|  | Fionidae Gray, 1857 |  | *Tenellia melanobrachia* (Bergh, 1874) | CAS167974b | Papua New Guinea | KY1284662 | KY129077 | KY128868 |
|  | Fionidae Gray, 1857 |  | *Tenellia melanobrachia* (Bergh, 1874) | CAS177298 | Philippines | KY1284663 | KY129078 | KY128869 |
|  | Fionidae Gray, 1857 |  | *Tenellia melanobrachia* (Bergh, 1874) | CAS177299 | Philippines | KY1284664 | KY129079 | KY128870 |
|  | Fionidae Gray, 1857 | *Phestilla minor* Rudman, 1981 | *Tenellia minor* | GB |  | - | DQ417311 | DQ417262 |
|  | Fionidae Gray, 1857 | *Phestilla sibogae* | *Tenellia lugubris* (Bergh, 1870) | GB |  | - | DQ417290 | DQ417244 |
|  | Fionidae Gray, 1857 | *Phestilla* sp. 3 | *Tenellia* sp. 3 | CAS177518 | Philippines | KY1284665 | KY129080 | KY128871 |
|  | Fionidae Gray, 1857 | *Phestilla* sp. A | *Tenellia* sp. L | CAS179523 | Steinhart Aquarium | KY1284659 | KY129074 | KY128865 |
|  | Fionidae Gray, 1857 | *Tenellia adspersa* (Nordmann, 1845) |  | CAS184191 | New Hampshire | KY1284668 | KY129085 | KY128876 |
|  | Fionidae Gray, 1857 |  |  | GNM9011 | Gotland, Sweden | - | KY129084 | KY128875 |
|  | Fionidae Gray, 1857 | *Tergipes antarcticus* Pelseneer, 1903 | *“Tergipes antarcticus”* Pelseneer, 1903 | GB |  | - | GU227106 | - |
|  | Fionidae Gray, 1857 | *Tergipes tergipes* (Forsskål in Niebuhr, 1775) |  | CAS182699 | Maine | KY1284670 | KY129087 | KY128878 |
|  | Fionidae Gray, 1857 |  |  | CAS183940 | Maine | KY1284671 | KY129088 | KY128879 |
|  | Fionidae Gray, 1857 |  |  | CAS184192 | New Hampshire | KY1284672 | KY129089 | KY128880 |
|  | Fionidae Gray, 1857 |  |  | WS3463 | Barents Sea | KY1284673 | KY129090 | KY128881 |
|  | Fionidae Gray, 1857 |  |  | WS3464 | Barents Sea | KY1284674 | KY129091 | KY128882 |
|  | Fionidae Gray, 1857 |  |  | WS3465 | Barents Sea | KY1284675 | KY129092 | KY128883 |
|  | Fionidae Gray, 1857 |  |  | GB |  | KJ434098 | KJ434080 | - |
|  | Fionidae Gray, 1857 |  |  | GB |  | KJ434099 | KJ434081 | - |
|  | Fionidae Gray, 1857 |  |  | GB |  | KJ434099 | - | - |
|  | Fionidae Gray, 1857 |  |  | GB |  | KJ434098 | KJ434080 | - |
|  | Fionidae Gray, 1857 |  |  | GB |  | KJ434094 | KJ434076 | KJ434063 |
|  | Fionidae Gray, 1857 |  |  | GB |  | KJ434088 | KJ434072 | KJ434057 |
|  | Fionidae Gray, 1857 |  |  | GB |  | KJ434082 | KJ434067 | KJ434052 |
|  | Fionidae Gray, 1857 | *Tergipes* sp. | *Tergiposacca longicerata*  gen. nov. | CAS177605 | Philippines | KY1284669 | KY129086 | KY128877 |
|  | Fionidae Gray, 1857 | *Trinchesia lenkae* Martynov, 2002 | *Tenellia lenkae* (Martynov, 2002) | AC17-19 | Sea of Japan | KY1284676 | KY129093 | KY128884 |

* Called as *Subcuthona pallida* in Gosliner *et al.* (2008)

** Called as *Cuthona* sp. 13 in Pittman and Fiene, 2014
