# Supplementary figures and images for: A Radical Solution: The Phylogeny of the Nudibranch Family Fionidae
Source: PLoS One. 2016 Dec 15;11(12):e0167800. doi: 10.1371/journal.pone.0167800 (PMC5158052; doi:10.1371/journal.pone.0167800)

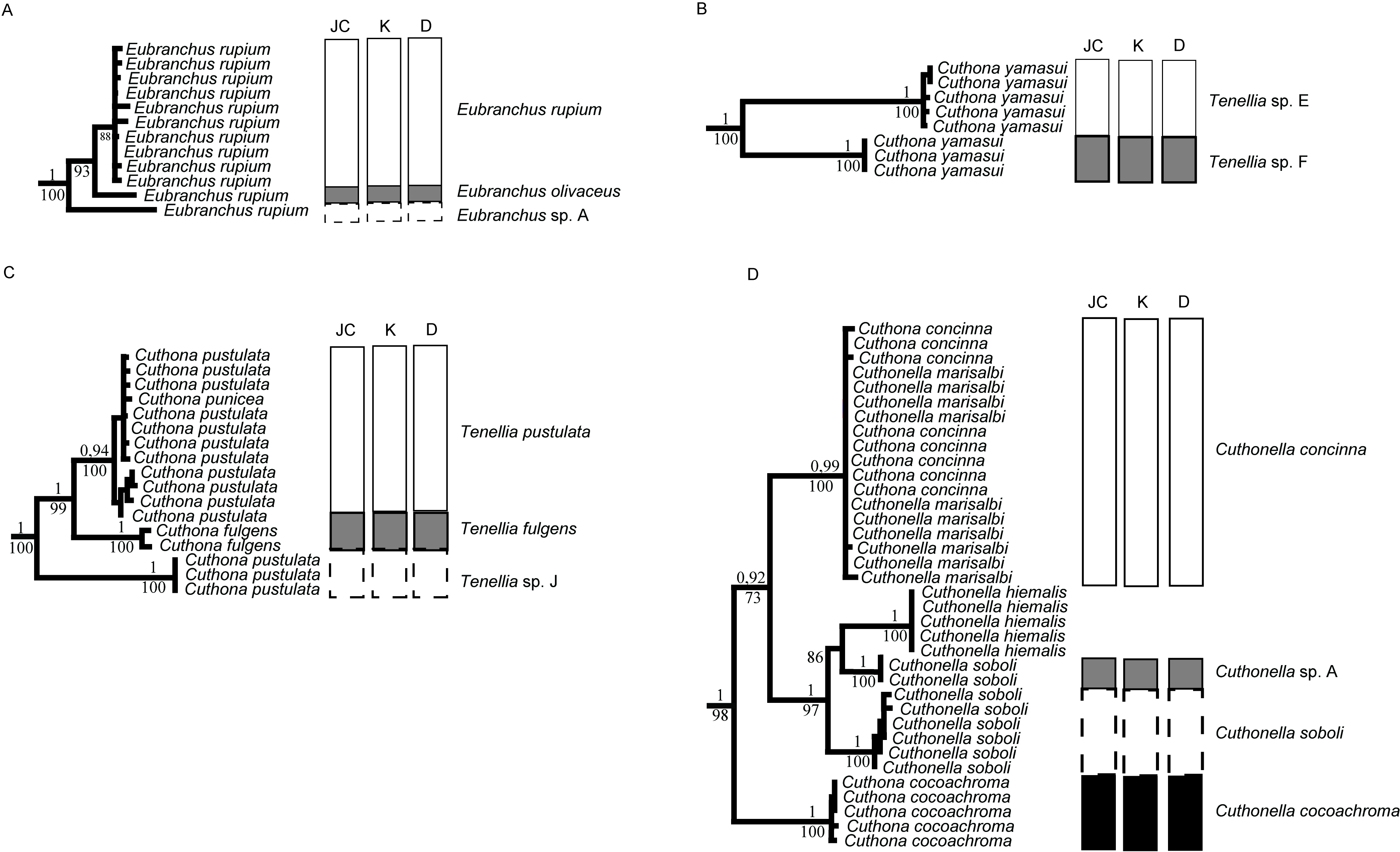

Supplement: S4 Fig — Trees were extracted from Fig 1. Rectangles represent the groups found by ABGD. (A) “Eubranchus rupium”. (B) “Cuthona yamasui”. (C) Cuthona pustulata and Cuthona punicea. (D) Cuthona concinna, Cuthonella marisalbi, Cuthonella soboli and Cuthona cocoachroma. Abbreviations: JC, Jukes-Cantor; K, Kimura; and D, Simple distance. (TIF) [file pone.0167800.s005.tif]
