# Supplementary material for: A Radical Solution: The Phylogeny of the Nudibranch Family Fionidae
Source: PLoS One. 2016 Dec 15;11(12):e0167800. doi: 10.1371/journal.pone.0167800 (PMC5158052; doi:10.1371/journal.pone.0167800)
Supplement: S1 Appendix — (DOCX) [file pone.0167800.s006.docx]

**Protocol followed by Kristen Cella and Terrence M. Gosliner**

Total genomic DNA was extracted from a tissue sample from each specimen using the Qiagen DNeasy Blood and Tissue Kit (Valencia, CA). In most cases, approximately 10 mg of tissue was taken from the posterior end of the foot. For very small specimens (<5 mm), most of or the entire specimen was used in the extraction process. All cerata were removed prior to the extraction process to prevent possible contamination of food in the digestive tracts. For each sample, three gene fragments were amplified using the following primers:

| 16S arL | CGCCTGTTTAACAAAAACAT | Palumbi *et al.*, 1996 |
| --- | --- | --- |
| 16S R | CCGRTYTGAACTCAGCTCACG | Palumbi *et al.*, 1996 |
| LCO 1490 | GGTCAACAAATCATAAAGATATTGG | Folmer *et al*., 1994 |
| HCO 2198 | TAAACTTCAGGGTGACCAAAAATCA | Folmer *et al.*, 1994 |
| COIH-2 | TAYACYTCRGGATGMCCAAAAAAT CA |  |
| H3 AF | ATGGCTCGTACCAAGCAGACVGC | Colgan *et al*., 1998 |
| H3 AR | ATATCCTTRGGCATRATRGTGAC | Colgan *et al*., 1998 |

All PCR amplifications were carried out in 25 μl reactions containing 1 μl of genomic DNA template, 2.5 μl of 10x PCR buffer, 0.5 μl dNTPs (10 μM stock), 1.5 μl MgCl2 (25 μM stock), 0.2 μl each primer (25 μM stock), 0.5 μl Taq- Apex (1.25 units/mL) and 18.6 μl of de-ionized water (ddH20). Additionally, 1 μl of bovine serum albumin (BSA) was added to each reaction. All reactions were run on either a Bio-Rad MyCycler™ or C1000™ thermal cycler. H3 fragments were amplified using the following parameters: initial denaturation at 94°C for 3 minutes, 35 cycles of 94°C for 35 seconds, 50- 55°C for 1 minute and 72°C for 1 minute 15 seconds, followed by a final extension at 72°C for 2 minutes. The COI amplification began with an initial denaturation at 94°C for 3 minutes, 40 cycles of 94°C for 30 seconds, 50- 56°C for 30 seconds, and 72°C for 1 minute, followed by a final extension at 72°C for 5 minutes. 16S amplification was performed with an initial denaturation at 94°C for 3 minutes, 39 cycles of 94°C for 30 seconds, 50-55°C for 30 seconds and 72°C for 1 minute, followed by a final extension at 72°C for 5 minutes. Successful PCR products were purified with ExoSap-IT™ (USB, Affymetrix, Fremont, CA) following standard protocol. In situations where amplification produced a double band, a gel excision purification method was employed. These PCR products were electropohresed on a 1% SeaPlaque™ Agarose (Lonza. Rockland, ME) gel stained with ethidium bromide in 1% TAE buffer. Targeted bands were excised from the gel and then purified with Agar*ACE*™ (Promega) using standard protocol. Cleaned PCR products were immediately cycle-sequenced in 10 μl reactions using BigDye® Terminator v3.1 (Applied Biosystems). Each reaction contained 1-2 μl of cleaned PCR product, 1.63 μl of 5X sequencing buffer, 0.75 μl of BigDye, 0.5 μl of primer (10 μM stock) and 5.13-6.13 μl of ddH20. The following parameters were used in all cycle sequencing reactions: initial denaturation step at 95°C for 1 minute, 15 cycles of 95°C for 10 seconds, 55°C for 5 seconds and 60°C for 1 minute and 15 seconds, then 5 cycles of 95°C for 10 seconds, 55°C for 5 seconds and 60°C for 1 minute 30 seconds, and finally 5 cycles of 95°C for 10 seconds, 55°C for 5 seconds and 60°C for 2 minutes. The same primers from the PCR reactions were used in the sequencing process with the exception of one COI primer. The HCO2198 primer did not work in several cases and a specially designed degenerate primer (COIH-2) was used instead. Cycle sequencing products were then precipitated by adding 2.5 μl of EDTA to each sample. The precipitates were then alternatively centrifuged and washed, first with 30 μl of 100% EtOH and then with 60 μl of 70% EtOH. Pellets were dried, re-suspended in 10 μl of formamide and denatured at 96°C for 2 minutes. Finally, all samples were sequenced in both directions on the ABI 3130xl Genetic Analyzer (Applied Biosystems) in the Center for Comparative Genomics at the California Academy of Sciences.

**Protocol followed by Leila Carmona**

Extraction, amplification, purification and sequencing of portions of the COI, 16S rRNA and H3 genes followed the methods described in Carmona et al. (2013). Sequence reactions were run on a 3730XL DNA sequencer (Applied Biosystems).

**Protocol followed by Irina Ekimova, Anton Chichvarkhin, and Dmitri Schepetov**

DNA was extracted from small pieces of foot tissue using Diatom™ DNA Prep 100 kit by Isogene according to the manufacturer’s protocol. Extracted DNA was used as a template for amplification of partial cytochrome *c* oxidase subunit I (COI), 16S rRNA (16S) and Histone 3 (H3).

For each sample, three gene fragments were amplified using the following primers:

| 16S arL | CGCCTGTTTAACAAAAACAT | Palumbi *et al.*, 1991 |
| --- | --- | --- |
| 16S R | CCGRTYTGAACTCAGCTCACG | Puslednik & Serb, 2008 |
| LCO 1490 | GGTCAACAAATCATAAAGATATTGG | Folmer *et al*., 1994 |
| HCO 2198 | TAAACTTCAGGGTGACCAAAAAATCA | Folmer *et al.*, 1994 |
| H3 AF | ATGGCTCGTACCAAGCAGACVGC | Colgan *et al*., 1998 |
| H3 AR | ATATCCTTRGGCATRATRGTGAC | Colgan *et al*., 1998 |

Polymerase chain reaction amplifications were carried out in a 20-µL reaction volume, included 4 µL of 5x Screen Mix by Eurogen Lab, 0.3 µL of each primer (10 µM stock), 1 µL of genomic DNA and 14,4 µL of sterile water. The amplification of COI was performed with an initial denaturation for 5 min at 95^o^C followed by 35 cycles of 15 s at 95^o^C (denaturation), 30 s at 45^o^C (annealing temperature) and 45 s at 72^o^C with a final extension of 7 min at 72^o^C. The 16S and H3 amplification began with an initial denaturation for 5 min at 95 ^o^C followed by 40 cycles of 15 s at 95 ^o^C (denaturation), 15 s at 52^o^C (annealing temperature), 30 s at 72 ^o^C with a final extension of 7 min at 72 ^o^C. Sequencing for both strands proceeded with the *Big Dye v3.1* sequencing kit by Applied Biosystems. Sequencing reactions were analyzed using ABI 3500 Genetic Analyzer (Applied Biosystems) in N.K. Koltsov Institute of Developmental Biology (Moscow, Russia).

BIBLIOGRAPHY

Carmona, L., Pola, M., Gosliner, T.M., Cervera JL (2013) A Tale That Morphology Fails to Tell: A Molecular Phylogeny of Aeolidiidae (Aeolidida, Nudibranchia, Gastropoda). PLoS One 8:e63000. doi:10.1371/journal.pone.0063000. doi: doi:10.1371/journal.pone.0063000

Colgan D, McLauchlan A, Wilson GDF, Livingston SP, Edgecombe GD, et al. (1998) Histone H3 and U2 snRNA DNA sequences and arthropod molecular evolution. Aust J Zool 46: 419–437.

Colgan, D. J., Ponder, W. F., Eggler, P. E. 1999. Gastropod evolutionary rates and phylogenetic relationships assessed using partial 28S rDNA and histone H3 sequences. Zool. Scripta 58, 116-123.

Folmer, O., Black, M., Hoeh, W., Lutz, R., Vrijenhoek, R. 1994. DNA primers for amplification of mitochondrial cytochrome *c* oxidase subunit I from diverse metezoan invertebrates. Mol. Mar. Biol. Biotechnol. 3, 294-299.

Palumbi SR, Martin A, Romano S, Owen MacMillan W, Stice L, et al. (1991) The Simple Fool’s Guide to PCR. Department of Zoology, University of Hawaii, Honolulu. 45 p.

Palumbi, S., Martin, A., Romano, S. McMillan, W. O., Stice, L., Grabowski, G. 1996. The simple fool’s guide to PCR, version 2.0. Hawaii: Department of Zoology and Kewalo Marine Laboratory, University of Hawaii.

Puslednik L., Serb J.M. 2008. Molecular phylogenetics of the Pectinidae (Mollusca: Bivalvia) and effect of increased taxon sampling and outgroup selection on tree topology // Molecular Phylogenetics and Evolution. Vol.48. P.1178–1188.
